# Persistence of viable opportunistic pathogens in a multi-stage natural wastewater treatment system

**DOI:** 10.1371/journal.pone.0354338

**Published:** 2026-07-27

**Authors:** Hazem Aqel, Husni Farah, Ramy Fodah, Naif Sannan, Ola Surakhi

**Affiliations:** 1 Basic Medical Sciences Department, College of Medicine, Al-Balqa Applied University, Salt, Jordan; 2 Medical Laboratory Sciences Department, Al-Ahliyya Amman University, Amman, Jordan; 3 Clinical Laboratory Sciences Department, College of Applied Medical Sciences, King Saud bin Abdulaziz University for Health Sciences, Riyadh, Saudi Arabia; 4 Clinical Laboratory Sciences Department, College of Applied Medical Sciences, King Saud bin Abdulaziz University for Health Sciences, Jeddah, Saudi Arabia; 5 Artificial Intelligence Department, Faculty of Information Technology, American University of Madaba, Madaba, Jordan; Government College University, Faisalabad, PAKISTAN

## Abstract

Nature-based wastewater treatment systems are increasingly implemented to support water reuse in arid regions, yet their effectiveness in removing viable culturable opportunistic pathogens remains insufficiently characterized. In this exploratory, culture-based study, we assessed bacterial population dynamics across a five-stage natural wastewater treatment system (Wadi Hanifa, Riyadh, Saudi Arabia), tracking culturable bacteria from secondary-treated influent to sand-filtered effluent. Water samples were collected at five sequential treatment stages and analyzed for physicochemical parameters, total culturable bacterial abundance, bacterial diversity, taxonomic composition, and antimicrobial susceptibility of persistent isolates. Total culturable bacterial counts decreased by approximately 1.1 log^10^ CFU mL^‒1^ across the system, accompanied by an approximately 50% reduction in observed isolate richness. The fecal indicator *Escherichia coli* was detected only in upstream and intermediate stages (sampling locations L3.1-L3.3) and was absent from downstream samples. In contrast, the opportunistic pathogen *Klebsiella pneumoniae* was recovered across all five treatment stages and accounted for 44.4% (8/18) of all morphologically distinct isolates grown on the selected culture media. Turbidity declined by 71% along the treatment train and showed a strong positive correlation with bacterial richness (Kendall’s τ = 0.84, p = 0.038), although this exploratory correlation should be interpreted with caution given the small sample size (n = 5 stages). Phenotypic antimicrobial susceptibility testing revealed multidrug resistance (MDR) in 62.5% (5/8) of *K. pneumoniae* isolates, although all remained susceptible to amikacin and meropenem. Under the conditions examined, multi-stage natural wastewater treatment substantially reduced overall bacterial abundance and diversity but did not eliminate viable, multidrug-resistant *Klebsiella pneumoniae*. The discordance between fecal-indicator removal and opportunistic pathogen recovery highlights system-specific limitations of indicator-based monitoring for assessing microbial safety in wastewater reuse systems. Given the limited isolate number (n = 8 *K. pneumoniae*) and the absence of molecular resistance-gene characterization, broader claims about wastewater as a dissemination pathway for antimicrobial resistance cannot be drawn from these data. These findings are based on a single cross-sectional sampling event and should be considered hypothesis-generating rather than confirmatory.

## Introduction

Increasing global water scarcity, particularly in arid and semi-arid regions, has driven growing reliance on wastewater reuse as a strategic resource [1–3]. In the Kingdom of Saudi Arabia, which generates approximately 3.2 million m^3^ day^‒1^ of municipal wastewater, treated effluent is increasingly used for agricultural and landscape irrigation [4,5]. Nature-based treatment systems, such as constructed wetlands and vegetated bio-cell corridors, offer low-energy, cost-effective treatment for arid-region reuse applications [6–8].

The microbiological safety of reclaimed water is conventionally assessed using fecal indicator organisms, principally *Escherichia coli* and total coliforms. However, accumulating evidence suggests that the removal of fecal indicators does not reliably predict the fate of opportunistic pathogens [9,10]. Fecal indicators are enteric in origin and may respond differently to environmental stressors than non-enteric opportunistic organisms [11,12]. Recent surveillance studies have documented the persistence of clinically relevant pathogens, including *Klebsiella pneumoniae*, in treated wastewater and reclaimed-water systems across diverse geographic settings [13,14].

*Klebsiella pneumoniae* is of particular concern in this context. Classified as a priority pathogen by the WHO, it is capable of causing urinary tract infections, pneumonia, and bloodstream infections, and is an important reservoir of antimicrobial resistance (AMR) determinants, including extended-spectrum β-lactamases (ESBLs) and carbapenemases [15–18]. Environmental strains have been shown to exhibit resistance profiles comparable to clinical isolates, raising concerns about dissemination via reclaimed-water irrigation [19,20].

The Wadi Hanifa natural wastewater treatment corridor in Riyadh, Saudi Arabia, represents one of the largest nature-based treatment systems in the Middle East, processing approximately 300,000 m^3^ day^‒1^ of secondary-treated municipal wastewater through sequential bio-cells and sand filtration before discharge for landscape irrigation [6]. Despite its scale and public-health relevance, the persistence of viable opportunistic pathogens across the Wadi Hanifa treatment stages has not previously been characterized using culture-based methods that confirm viability.

We hypothesized that multi-stage natural treatment would reduce total culturable bacterial abundance and diversity but that certain opportunistic pathogens-particularly *K. pneumoniae*-would persist through the complete treatment train owing to their environmental adaptability, capsule production, and resistance to physicochemical stressors. We further hypothesized that fecal-indicator removal would not accurately predict the fate of these organisms.

## Materials and methods

### Study area and sampling design

Sampling was conducted along a 4.8-km section of the Wadi Hanifa natural wastewater treatment corridor in northwest Riyadh, Saudi Arabia (24°34′ N, 46°43′ E), in November 2015. The Wadi Hanifa system receives approximately 300,000 m^3^ day^‒1^ of secondary-treated municipal wastewater and employs sequential natural treatment processes over a hydraulic residence time of 4–6 days. Sampling was performed in November to represent stable dry-season operating conditions; seasonal variation in temperature, organic loading, and flow rates may influence bacterial community composition, and the findings should be interpreted accordingly.

Although the samples were collected in November 2015, the operational configuration, hydraulic residence time, vegetation regime, and effluent-reuse pattern of the Wadi Hanifa corridor have remained substantively unchanged since that time, as confirmed by recent operational reports of the Riyadh municipal water authority. Culture-based viability data of this granularity remain scarce for arid-region nature-based treatment systems, and the dataset therefore retains baseline relevance for current reuse-safety assessment.

Access to the Wadi Hanifa treatment corridor was granted under a research agreement with King Abdullah International Medical Research Centre (KAIMRC; reference KAIMRC/RFC/0092/17). The final sand-filtered effluent (L3.5) is currently used primarily for landscape irrigation and green-belt maintenance within the Riyadh metropolitan area; no terminal chlorination step is applied before this discharge.

**Five sampling locations** representing sequential treatment stages were selected: **L3.1** (Raw influent) — inlet receiving secondary-treated municipal wastewater; **L3.2** (Post-screening) – after coarse screening and grit removal, ~ 500 m downstream; **L3.3** (Bio-cell 1) – first vegetated bio-cell dominated by *Phragmites australis*, ~ 1.2 km downstream; **L3.4** (Bio-cell 2) – second bio-cell with established aquatic fauna and algal communities, ~ 2.8 km downstream; **L3.5** (Final effluent) – post-sand filtration, ~ 4.8 km downstream.

**Terminology Note.** Throughout this manuscript, “culturable” refers to bacteria recoverable under the specific media and incubation conditions used in this study. “Viable” is used in the conventional sense of having metabolic activity and the potential for growth; however, culturability is considered a proxy for viability rather than a perfect synonym, as the viable-but-non-culturable (VBNC) state is well documented for many bacterial taxa, including Klebsiella pneumoniae, and is not captured by the culture-based approach used here. “Culture-based” describes the overall methodological framework, and findings should be interpreted with the understanding that VBNC cells are not enumerated.

### Sample collection

Water samples (1 L) were collected in sterile, screw-cap polypropylene bottles at each of the five sampling locations. Samples were transported to the laboratory on ice and processed within 4 hours of collection. Triplicate samples were collected at each location to enable calculation of means and standard deviations.

### Physicochemical analysis

Physicochemical parameters were measured in situ at each sampling location using a calibrated YSI Pro Plus multiparameter probe. Parameters measured included pH, temperature (°C), dissolved oxygen (DO; mg L^‒1^), turbidity (NTU), and electrical conductivity (EC; µS cm^‒1^). Three independent replicate readings per parameter were taken after a ~ 2-min stabilization period, with ~2 m spacing between replicate measurement positions. Raw triplicate measurements are provided in Supporting Table S3 in S1 File.

### Bacterial isolation and enumeration

Total culturable bacterial counts were determined by spread-plating serial dilutions (10^‒1^ to 10^‒6^) onto blood agar (BA) and MacConkey agar (MAC) plates in triplicate. Plates were incubated at 37°C for 24–48 h. Morphologically distinct colony types were counted and selected for further characterization. All colony counts were performed by two independent operators; counts were accepted when the coefficient of variation between triplicates was ≤ 15%. Raw triplicate counts are provided in Supporting Table S1 in S1 File.

### Phenotypic and biochemical characterization

Morphologically distinct isolates were subjected to Gram staining and standard biochemical characterization using API 20E strips (bioMérieux, Marcy-l’Étoile, France) according to the manufacturer’s instructions. API 20E profiles were read at 24 h and 48 h and interpreted using the API® web database. Hierarchical clustering of biochemical profiles was performed to assess phenotypic similarity among isolates (Supporting Table S2 in S1 File). Isolate selection for further molecular characterization was based on frequency of recovery, phenotypic distinctiveness, and clinical relevance of the presumptive identification.

### DNA extraction

Genomic DNA was extracted from overnight cultures using the boiling method: cells were resuspended in sterile distilled water, boiled for 10 min, centrifuged at 13,000 × g for 5 min, and the supernatant used as template. DNA concentration and purity were assessed by NanoDrop spectrophotometry (A260/A280 ≥ 1.8). The guanine‑plus‑cytosine (G+C) content was determined by the thermal denaturation method as described by Marmur and Doty [21].

### Detection of 16S rRNA using PCR

16S rRNA gene amplification was performed using universal primers KP_16S F1 (NM3_F) (5′- ATGTCGCAAGACCAGAGTGG −3′) and KP_16S R1 (NM3_R) (5′- GCACAACCTCCAAATCGACA −3′). PCR conditions: 94°C for 3 min; 35 cycles of 94°C for 30 s, 58°C for 30 s, 72°C for 30 s; final extension 72°C for 10 min. Products (~657 bp) were resolved on 1.5% agarose gels.

### Antimicrobial susceptibility testing

Antimicrobial susceptibility of the eight *K. pneumoniae* isolates was determined by Kirby-Bauer disk diffusion on Mueller–Hinton agar, following CLSI M100 (33rd edition, 2023) guidelines [22]. Eight antimicrobial agents were tested: ampicillin (10 µg), amoxicillin-clavulanate (20/10 µg), ceftazidime (30 µg), cefotaxime (30 µg), ciprofloxacin (5 µg), trimethoprim-sulfamethoxazole (1.25/23.75 µg), amikacin (30 µg), and meropenem (10 µg). Zone diameters were measured in triplicate and interpreted as susceptible, intermediate, or resistant. Multidrug resistance was defined as resistance to ≥3 antimicrobial classes. Raw zone-of-inhibition measurements are provided in Supporting Table S4 in S1 File.

### Statistical analysis

All analyses were performed using R version 4.3.2 [23]. Bacterial diversity at each treatment stage was quantified using isolate richness (S), the Shannon-Wiener index (H′), Simpson’s diversity index (1 – D), and Pielou’s evenness (J′). Trends in culturable bacterial abundance across treatment stages were evaluated using linear regression. Given the ordinal nature of the treatment stages and the small sample size, Cochran-Armitage exact tests were used to detect monotonic trends in categorical variables. All statistical analyses presented here are exploratory and intended to identify trends consistent with biological mechanisms, rather than to provide definitive inferential conclusions. Associations between physicochemical parameters and bacterial richness were examined using Kendall’s τ rank correlation. Differences in Gram-type composition across treatment stages were evaluated using Fisher’s exact test. Statistical significance was defined at α = 0.05; all p-values are reported as exploratory.

### Quality assurance and quality control

All culture media were prepared from commercially certified dehydrated powders and quality-controlled using ATCC reference strains (*E. coli* ATCC 25922, *K. pneumoniae* ATCC 13883) prior to use. Positive and negative controls were included in each PCR run. Field blanks (sterile distilled water processed identically to samples) were included at each sampling visit; no contamination was detected. All glassware and sampling equipment were sterilized by autoclaving (121°C, 15 min, 15 psi) prior to use.

### Ethical considerations

This study involved collection of environmental water samples from a managed public treatment waterway. No human subjects, animals, or personal data were involved. Approval was granted by the KAIMRC Institutional Review Board (reference KAIMRC/RFC/0092/17). No additional ethical approval was required.

## Results and discussion

### Physicochemical changes across treatment stages

Progressive improvements in physicochemical quality were observed across the five treatment stages (Table 1; raw triplicate data in Supporting Table S3 in S1 File). Turbidity declined from 28.4 NTU at L3.1 to 8.2 NTU at L3.5, representing a 71% reduction. Dissolved oxygen increased progressively from 3.1 mg L^‒1^ at L3.1 to 7.8 mg L^‒1^ at L3.5, consistent with phytoremediation activity in the bio-cells. pH remained within a narrow circumneutral range (7.2–7.9) throughout the system. Electrical conductivity declined modestly (1,280–1,045 µS cm^‒1^), suggesting limited ion removal by the natural treatment processes. Temperature (22.4–24.1°C) was relatively stable across stages, consistent with sampling in the November dry season.

**Table 1:** Physicochemical parameters across sequential treatment stages (mean ± standard deviation from triplicate measurements).

Stage	Description	pH	Temp (°C)	DO (mg/L)	Turbidity (NTU)	EC (µS/cm)
L3.1	Raw Influent	7.4 ± 0.1	24.8 ± 0.3	4.8 ± 0.2	42 ± 3	1820 ± 40
L3.2	Post-Screening	7.5 ± 0.1	25.0 ± 0.2	5.4 ± 0.2	35 ± 2	1805 ± 35
L3.3	Bio-cell 1	7.6 ± 0.1	25.2 ± 0.3	6.4 ± 0.3	24 ± 2	1780 ± 30
L3.4	Bio-cell 2	7.7 ± 0.1	25.4 ± 0.2	7.2 ± 0.2	17 ± 1	1755 ± 25
L3.5	Final Effluent	7.8 ± 0.1	25.5 ± 0.2	7.9 ± 0.3	12 ± 1	1740 ± 20

Values are mean ± standard deviation from triplicate replicate measurements at each sampling location (n = 3). DO = dissolved oxygen; EC = electrical conductivity; NTU = nephelometric turbidity units; Temp = temperature; °C = degrees Celsius; mg/L = milligrams per liter; μS/cm = microsiemens per centimeter.

### Reduction in culturable bacterial load

Total culturable bacterial counts declined by approximately 1.1 log^10^ CFU mL^‒1^ across the treatment system (Table 2 and Supporting Table S1 in S1 File), with the greatest single-stage reduction occurring between Bio-cell 1 (L3.3) and Bio-cell 2 (L3.4). Linear regression revealed a consistent monotonic decreasing trend across treatment stages (R^2^ ≥ 0.96; p < 0.001, exploratory). Because only five treatment stages were sampled, this high R^2^ value should be interpreted cautiously, as regression models fitted to n = 5 data points carry an elevated risk of overfitting; the result is best regarded as an indicator of a consistent monotonic trend rather than a precise quantitative relationship.

**Table 2:** Reduction in culturable bacterial counts across treatment stages.

Stage	Description	BA Count (CFU mL^‒1^) Mean ± SD	MacConkey Count (CFU mL^‒1^) Mean ± SD
L3.1	Raw Influent	(4.2 ± 0.4) × 10⁴	(5.8 ± 0.5) × 10⁴
L3.2	Post-Screening/Grit Removal	(3.1 ± 0.3) × 10⁴	(4.4 ± 0.4) × 10⁴
L3.3	First Bio-cell (Phragmites)	(1.9 ± 0.2) × 10⁴	(2.7 ± 0.3) × 10⁴
L3.4	Second Bio-cell (Fish/Algae)	(8.0 ± 0.8) × 10³	(1.1 ± 0.1) × 10⁴
L3.5	Final Sand-Filtered Effluent	(3.5 ± 0.3) × 10³	(4.5 ± 0.4) × 10³

Values are mean ± standard deviation from triplicate plate counts (n = 3 plates per dilution). BA = blood agar; MAC = MacConkey agar; CFU = colony-forming units; mL = milliliter. The highest dilution yielding countable colonies was used for enumeration in each case.

Despite this reduction, the overall log^10^ decrease remains well below the ≥ 5-log pathogen-removal targets recommended by the WHO for unrestricted irrigation reuse [24]. **It is important to emphasize that WHO ≥ 5-log targets refer to pathogen-specific reductions** (e.g., for rotavirus, *Cryptosporidium*, and selected bacterial pathogens, generally evaluated at the unit-process level), **not** to total culturable heterotrophic bacterial counts. The comparison here is provided solely for contextual scale; total CFU reduction is **not** a direct equivalent of pathogen-specific log-removal credits. These findings suggest that while natural treatment substantially improves microbiological quality, it may be insufficient as a sole barrier for pathogen-risk control in reuse applications, and terminal disinfection should be considered.

### Shifts in viable bacterial composition and persistence of Klebsiella pneumoniae

Culture-based profiling of 18 morphologically distinct isolates revealed pronounced shifts in bacterial composition along the treatment train (Table 3). *Escherichia coli* was detected only up to Bio-cell 1 (L3.3) and was absent from downstream stages, consistent with effective removal of fecal indicators. In contrast, *Klebsiella pneumoniae* persisted across all five treatment stages and accounted for 44.4% (8/18) of all characterized isolates.

**Table 3:** Presumptive identification of bacterial isolates via API 20E biochemical profiling.

Isolate IDs	API Code	Identification	Confidence (%)	n	% of Total	Stages Present
1,4,9,10,13,15,16,17	5215773	*Klebsiella pneumoniae*	92–97	8	44.4	All (L3.1–L3.5)
5,7,14	5144552	*Escherichia coli*	89–94	3	16.7	L3.1–L3.3 only
6,11	5347125	Enterobacter cloacae complex	86–91	2	11.1	L3.3, L3.4
2,8	5375573	*Proteus mirabilis*	85–88	2	11.1	L3.2, L3.4
3,12,18	Various	Other Gram-positive rods	80–85	3	16.7	Variable

Presumptive identification based on API 20E biochemical profiles (bioMérieux, Marcy-l’Étoile, France). API codes are 7-digit numerical profiles; identification confidence based on manufacturer’s database (≥85% considered acceptable). n = number of isolates; % = percentage of total isolates characterized; L3.1-L3.5 = sampling locations along treatment train (influent to final effluent). Isolate IDs correspond to morphologically distinct colonies selected from blood agar and MacConkey agar plates.

The hierarchical clustering of biochemical profiles (Supporting Table S2 in S1 File) showed that all eight *K. pneumoniae* isolates-recovered across the full treatment train-grouped within a tight metabolic cluster distinct from *E. coli*, *Enterobacter*, and *Proteus* isolates, indicating phenotypic coherence of the persistent *K. pneumoniae* population and consistency of identification across stages.

This divergence underscores a system-specific limitation of indicator-based monitoring. The absence of *E. coli* would conventionally be interpreted as adequate microbial safety; however, the continued recovery of viable *K. pneumoniae* across all treatment stages demonstrates that clinically relevant opportunistic pathogens may persist even when fecal-indicator thresholds are met. Possible explanations include: (i) use of non-selective BA and MAC agar rather than selective chromogenic media, which may have led to underestimation of *E. coli* in downstream samples; (ii) the known environmental persistence of *K. pneumoniae*, facilitated by capsule formation, biofilm production, and metabolic versatility [18]; and (iii) the typically lower environmental stress tolerance of *E. coli* compared with non-enteric opportunistic pathogens under aerobic, low-turbidity conditions. Similar discrepancies have been documented in wastewater and reclaimed-water systems [13,25–28].

### Antimicrobial resistance profiles of persistent *Klebsiella pneumoniae*

Phenotypic antimicrobial susceptibility testing of the eight *K. pneumoniae* isolates revealed multidrug resistance (MDR; resistance to ≥3 antimicrobial classes) in 62.5% (5/8) of isolates (Table 4). Universal resistance to ampicillin was observed, consistent with the intrinsic AmpC-like β-lactamase of *K. pneumoniae*. High resistance rates were observed for ceftazidime (75%), amoxicillin-clavulanate (62.5%), and trimethoprim-sulfamethoxazole (62.5%), suggesting possible extended-spectrum β-lactamase (ESBL) or plasmid-mediated resistance. All isolates retained susceptibility to amikacin and meropenem, indicating that last-resort carbapenems remained effective against these isolates.

**Table 4:** Antimicrobial susceptibility patterns of *Klebsiella pneumoniae* isolates (n = 8).

Antimicrobial Agent (Class)	Resistant (%)	Intermediate (%)	Susceptible (%)
Ampicillin (Penicillin)	100	0	0
Amoxicillin-Clavulanate (β-lactam)	62.5	12.5	25
Ceftazidime (3rd-gen. Cephalosporin)	75	0	25
Gentamicin (Aminoglycoside)	37.5	12.5	50
Ciprofloxacin (Fluoroquinolone)	50	12.5	37.5
Trimethoprim-Sulfamethoxazole (Folate inhibitor)	62.5	0	37.5
Amikacin (Aminoglycoside)	0	0	100
Meropenem (Carbapenem)	0	0	100
MDR Phenotype (≥3 classes)	62.5 (5/8)	–	–

Antimicrobial susceptibility testing performed by Kirby-Bauer disk diffusion on Mueller-Hinton agar following CLSI M100 (33rd edition, 2023) guidelines. n = 8 K. pneumoniae isolates recovered across all treatment stages. MDR = multidrug resistance, defined as resistance to ≥3 antimicrobial classes. Percentages are based on n = 8 isolates. CLSI interpretive criteria: S = susceptible, I = intermediate, R = resistant. All isolates were tested in triplicate (raw zone-of-inhibition measurements provided in Supporting Table S4 in S1 File).

The recovery of viable MDR *K. pneumoniae* in the final sand-filtered effluent is consistent with recent reports suggesting that wastewater systems may act as environmental compartments where antimicrobial-resistant organisms can persist [18,29–34]. However, given the limited number of isolates (n = 8) and the absence of molecular characterization of resistance determinants in this study, broader claims regarding the role of this system as a dissemination pathway for antimicrobial resistance cannot be substantiated from the present dataset and would require dedicated genomic, longitudinal, and source-tracking investigations. Culture-based confirmation does, however, demonstrate that resistance phenotypes detected here correspond to viable organisms with environmental-persistence potential. Molecular characterization of resistance genes (e.g., *bla*SHV, *bla*TEM, *bla*CTX-M) and plasmid replicon typing would be needed to confirm the genetic basis of resistance in future studies. Raw zone-of-inhibition measurements for all eight isolates are provided in Supporting Table S4 in S1 File.

### Bacterial diversity, correlations with physicochemical parameters, and compositional stability

Observed isolate richness declined by approximately 50% across treatment stages (from S = 4 at L3.1 to S = 2 at L3.5; Cochran–Armitage exact test, p = 0.022; n = 5 stages, exploratory), accompanied by reductions in Shannon–Wiener (H′: 1.33 → 0.69) and Simpson (1 – D: 0.73 → 0.45) diversity indices (Table 5). Turbidity exhibited a strong positive rank correlation with bacterial richness (Kendall’s τ = 0.84, p = 0.038), supporting the role of particulate removal as a primary driver of microbial diversity loss. Because only five treatment stages were sampled, this correlation is exploratory; nonetheless, the result is consistent with the established role of particle-associated transport in governing microbial fate in constructed wetland and bio-cell systems [16,35–37].

**Table 5:** Bacterial diversity metrics across treatment stages.

Stage	Richness (S)	Shannon-Wiener Index (H′)	Simpson’s Diversity Index (1-D)	Evenness (J′)
L3.1	4	1.33	0.73	0.96
L3.2	4	1.30	0.72	0.94
L3.3	4	1.28	0.71	0.92
L3.4	3	0.95	0.58	0.86
L3.5	2	0.69	0.45	0.99

S = observed isolate richness (number of distinct morphotypes isolated per 500 mL sample). H’ = Shannon-Wiener diversity index. 1-D = Simpson’s diversity index. J’ = Pielou’s evenness (H’/ln S). Values are based on 18 morphologically distinct isolates characterized across five treatment stages. L3.1-L3.5 = sequential treatment stages from influent to final effluent.

Dissolved oxygen showed a moderate negative trend with bacterial richness (Kendall’s τ = −0.74, p = 0.062), while pH, temperature, and EC were not significantly correlated with diversity metrics (Table 6). These findings suggest that physical removal processes-particularly sedimentation and filtration of particle-associated bacteria-were the dominant drivers of bacterial diversity loss, rather than gradual changes in water chemistry.

**Table 6:** Kendall’s τ correlations between physicochemical parameters and bacterial richness (n = 5 treatment stages).

Parameter	Kendall’s τ	p-value	Interpretation
Turbidity	+0.84	0.038*	Strong positive correlation
Dissolved Oxygen	−0.74	0.062	Moderate negative trend (not significant)
pH	−0.68	0.089	Weak negative trend (not significant)
Temperature	−0.21	0.621	No correlation
EC	+0.47	0.234	No significant correlation

τ = Kendall’s rank correlation coefficient; p = p-value (two-tailed). n = 5 treatment stages. *Significant at α = 0.05. DO = dissolved oxygen; EC = electrical conductivity. All p-values are reported as exploratory given the small sample size. Interpretations are qualitative and intended to identify trends consistent with biological mechanisms.

Despite overall diversity reduction, the proportion of Gram-negative isolates remained statistically stable across all treatment stages (Fisher’s exact test, p = 0.637; Table 5). With such small numbers of isolates per stage, this non-significant result carries a high risk of Type II error and should not be interpreted as confirming true compositional stability. The recovery of Gram-negative taxa, including *K. pneumoniae*, across all stages further suggests that capsule formation, metabolic versatility, and environmental adaptability are more critical determinants of survival than cell-wall structure [17, 38].

Representative *K. pneumoniae* isolates from all five treatment stages were further characterized molecularly (Table 7). All isolates showed %G + C contents consistent with the species (57.1–57.5%) and produced the expected 16S rRNA amplicon, supporting their identification. Detailed raw data for these molecular analyses are provided in Supporting Table S4 and Figure S1 in S1 File.

**Table 7:** Molecular characterization of representative *Klebsiella pneumoniae* isolates (n = 5, one from each treatment stage).

Isolate ID	Treatment Stage	%G + C Content (Thermal Denaturation)	16S rRNA PCR Result (Product Size)	Confirmation
1	L3.1 Raw Influent	57.2 ± 0.3	Positive (≈657 bp)	*Consistent with K. pneumoniae*
4	L3.2 Post-Screening	57.4 ± 0.2	Positive (≈657 bp)	*Consistent with K. pneumoniae*
9	L3.3 Bio-cell 1	57.1 ± 0.4	Positive (≈657 bp)	*Consistent with K. pneumoniae*
13	L3.4 Bio-cell 2	57.3 ± 0.2	Positive (≈657 bp)	*Consistent with K. pneumoniae*
15	L3.5 Final Effluent	57.5 ± 0.3	Positive (≈657 bp)	*Consistent with K. pneumoniae*

%G + C = guanine-plus-cytosine content determined by thermal denaturation method [21]. Values are mean ± standard deviation from triplicate thermal denaturation measurements. bp = base pairs. PCR = polymerase chain reaction. All five isolates confirmed as consistent with *Klebsiella pneumoniae* based on %G + C content (expected range: 57–58%) and 16S rRNA PCR amplicon size (expected size: ~ 657 bp). See Supporting Table S4 and Figure S1 in S1 File for raw agarose gel image.

### Implications for wastewater reuse monitoring and public health

The contrasting fate of *E. coli* and *K. pneumoniae* within this system (described in the preceding sub-section) demonstrates that fecal-indicator compliance does not guarantee removal of opportunistic pathogens under the conditions examined. Recent reviews and policy analyses increasingly recognize the limitations of indicator-only frameworks for reclaimed-water safety [11,15,24,26].

The recovery of viable MDR *K. pneumoniae* in the final effluent-which is used for landscape irrigation without terminal disinfection-suggests a potential exposure pathway that merits further risk quantification through quantitative microbial risk assessment (QMRA) [39,40], while acknowledging that the present cross-sectional dataset is not sufficient to characterize exposure or risk quantitatively.

These preliminary findings support calls to supplement routine indicator monitoring with targeted surveillance of clinically relevant opportunistic pathogens and to integrate terminal disinfection barriers into nature-based treatment systems when effluent reuse is intended. From a conceptual QMRA perspective, the recovery of viable MDR *K. pneumoniae* in irrigation-grade effluent suggests potential exposure pathways including aerosol inhalation during sprinkler irrigation, dermal contact during agricultural activity, and indirect ingestion through produce. Incorporating viability-based pathogen data into QMRA frameworks, together with dose-response models for opportunistic pathogens, may substantially improve the accuracy of health-risk estimates in arid-region reuse contexts [40]. Larger, longitudinal studies employing both culture-based and molecular approaches are needed to confirm and extend these observations.

## Conclusions

This exploratory culture-based investigation provides preliminary evidence that a multi-stage natural wastewater treatment system can substantially reduce overall bacterial load and diversity while permitting the persistence of viable, frequently multidrug-resistant *Klebsiella pneumoniae*. Under the conditions examined, the elimination of *E. coli* contrasted with the continued recovery of *K. pneumoniae* across all treatment stages, revealing a system-specific limitation of fecal-indicator-based monitoring for water-reuse safety assessment.

These findings are based on a single sampling event with a limited number of isolates (n = 18) and should be regarded as preliminary and hypothesis-generating. Although the samples were collected in November 2015, the operational configuration of the Wadi Hanifa corridor has remained stable, supporting the continuing relevance of the dataset; however, larger, multi-season studies with expanded isolate collections, molecular confirmation, and resistance-gene characterization are required to validate and generalize these observations. The results highlight the potential need to integrate targeted pathogen surveillance and additional treatment barriers, such as terminal disinfection, into nature-based systems to support sustainable and safe wastewater reuse in arid regions.

## Supporting information

S1 File S1 Table. Complete culturable bacterial counts (CFU mL^‒1^) across the five treatment stages, including mean ± SD from triplicate plate counts on blood agar (BA) and MacConkey agar (MAC). Table S2. Hierarchical clustering of the 18 bacterial isolates based on API 20E biochemical profiles. Groupings reflect similarity in metabolic traits and are consistent with presumptive taxonomic identification. Table S3. Raw triplicate physicochemical measurements across sequential treatment stages, underlying the means ± SD reported in Table 1. Instrument: YSI Pro Plus multiparameter probe (calibrated prior to each sampling event). Sampling date: November 2015; Wadi Hanifa treatment corridor, Riyadh, Saudi Arabia (24°34′ N, 46°43′ E). Table S4. Raw zone-of-inhibition measurements (mm) and CLSI interpretive categories for all eight K. pneumoniae isolates across the eight antimicrobial agents tested; raw agarose gel images of 16S rRNA PCR products for the five representative isolates; and individual API 20E reaction scores for all 18 isolates. Figure S1. Representative agarose gel electrophoresis images of 16S rRNA PCR products for K. pneumoniae isolates. (A) Lane 1: 100 bp DNA ladder; Lane 2: positive control (K. pneumoniae ATCC 13883); Lanes 3–7: five representative isolates from L3.1–L3.5; Lane 8: negative control (sterile water). Expected amplicon size: ~ 657 bp. (B) Confirmation gel showing PCR products from all eight K. pneumoniae isolates with DNA ladder. Gel conditions: 1.5% agarose, 100 V for 45 min, stained with ethidium bromide.
(DOCX)
