# Supplementary material for: Persistence of viable opportunistic pathogens in a multi-stage natural wastewater treatment system
Source: PLoS One. 2026 Jul 27;21(7):e0354338. doi: 10.1371/journal.pone.0354338 (PMC13405070; doi:10.1371/journal.pone.0354338)

**Supporting information**

**Table S1.** Complete culturable bacterial counts (CFU/mL) across the treatment stages.

| **Stage** | **Description** | **BA Rep 1 (CFU/mL)** | **BA Rep 2 (CFU/mL)** | **BA Rep 3 (CFU/mL)** | **BA Count (Mean ± SD)** | **MAC Rep 1 (CFU/mL)** | **MAC Rep 2 (CFU/mL)** | **MAC Rep 3 (CFU/mL)** | **MAC Count (Mean ± SD)** |
| --- | --- | --- | --- | --- | --- | --- | --- | --- | --- |
| L3.1 | Raw Influent | 46,000 | 38,000 | 42,000 | (4.2 ± 0.4) × 10⁴ | 63,000 | 55,000 | 56,000 | (5.8 ± 0.5) × 10⁴ |
| L3.2 | Post-Screening/Grit Removal | 34,000 | 28,000 | 31,000 | (3.1 ± 0.3) × 10⁴ | 48,000 | 40,000 | 44,000 | (4.4 ± 0.4) × 10⁴ |
| L3.3 | First Bio-cell (Phragmites) | 21,000 | 17,000 | 19,000 | (1.9 ± 0.2) × 10⁴ | 30,000 | 24,000 | 27,000 | (2.7 ± 0.3) × 10⁴ |
| L3.4 | Second Bio-cell (Fish/Algae) | 8,800 | 7,200 | 8,000 | (8.0 ± 0.8) × 10³ | 12,000 | 10,000 | 11,000 | (1.1 ± 0.1) × 10⁴ |
| L3.5 | Final Sand-Filtered Effluent | 3,800 | 3,200 | 3,500 | (3.5 ± 0.3) × 10³ | 4,900 | 4,100 | 4,500 | (4.5 ± 0.4) × 10³ |

*Note: SD: Standard Deviation estimated from triplicate plate counts. CFU: Colony Forming Units. BA = Blood Agar; MAC = MacConkey Agar. Raw triplicate values are underlying data for the means ± SD reported in Table 2 of the main manuscript.*

**Table S2.** Hierarchical clustering of isolates based on their biochemical profiles.

| Cluster | Isolate IDs (as per source) | Presumptive Identification | Key Shared Metabolic Traits | Stages Present |
| --- | --- | --- | --- | --- |
| **Cluster I** | 1,4,9,10,13,15,16,17 (and multiple entries in raw data) | *Klebsiella pneumoniae* | ONPG⁺, ADH⁺, LDC⁺, ODC⁺, UREA⁺, VP⁺, CIT variable, IND⁻ | All (L3.1–L3.5) |
| **Cluster II** | 5,7,14 (and 1/1/G BA2, 1/4/R Mac2, etc.) | *Escherichia coli* | ONPG⁺, LDC⁺, ODC⁻, CIT⁻, IND⁻, VP⁻, GLU⁺, MAN⁺ | L3.1–L3.3 only |
| **Cluster III** | 6,11 (and 1/1/G Mac1, 2/4/R BA2.2, etc.) | *Enterobacter cloacae* complex | ONPG⁺, ADH⁺, LDC⁺, ODC⁺, CIT⁺, VP⁺, IND⁻, INO⁻ | L3.3, L3.4 |
| **Cluster IV** | 2,8 (and 1/2/G BA2, 3/1 Mac3, etc.) | *Proteus mirabilis* | ONPG⁺, CIT⁻, H₂S⁻, UREA⁺, TDA⁺, IND⁻, VP⁻, GEL⁺ | L3.2, L3.4 |
| **Cluster V** | 3,12,18 (and various Gram-positive rods) | Other Gram-positive rods (e.g., *Staphylococcus*, *Erwinia*, *Pantoea*) | Variable; generally ONPG⁻ or weak, Gram-positive morphology | Variable across stages |

*Hierarchical grouping is based on euclidean distance of API 20E binary results (positive/negative). All eight K. pneumoniae isolates (Cluster I) formed a tight subcluster with >95% profile similarity. Full API 20E reaction matrices are available in Bacteria isolated from Wadi Hanifa.xlsx (Sheets 1-3).*

**Table S3.** Raw triplicate Physicochemical measurements across sequential treatment stage (underlying means ± SD reported in Table 1)

| **Stage** | **Parameter** | **Rep 1** | **Rep 2** | **Rep 3** | **Mean ± SD** |
| --- | --- | --- | --- | --- | --- |
| L3.1 | pH | 7.4 | 7.5 | 7.3 | 7.4 ± 0.1 |
|  | Temp (°C) | 24.8 | 25.1 | 24.5 | 24.8 ± 0.3 |
|  | DO (mg/L) | 4.8 | 5.0 | 4.6 | 4.8 ± 0.2 |
|  | Turbidity (NTU) | 42 | 45 | 39 | 42 ± 3 |
|  | EC (µS/cm) | 1820 | 1860 | 1780 | 1820 ± 40 |
| L3.2 | pH | 7.5 | 7.6 | 7.4 | 7.5 ± 0.1 |
|  | Temp (°C) | 25.0 | 25.2 | 24.8 | 25.0 ± 0.2 |
|  | DO (mg/L) | 5.4 | 5.6 | 5.2 | 5.4 ± 0.2 |
|  | Turbidity (NTU) | 35 | 37 | 33 | 35 ± 2 |
|  | EC (µS/cm) | 1805 | 1840 | 1770 | 1805 ± 35 |
| L3.3 | pH | 7.6 | 7.7 | 7.5 | 7.6 ± 0.1 |
|  | Temp (°C) | 25.2 | 25.5 | 24.9 | 25.2 ± 0.3 |
|  | DO (mg/L) | 6.4 | 6.7 | 6.1 | 6.4 ± 0.3 |
|  | Turbidity (NTU) | 24 | 26 | 22 | 24 ± 2 |
|  | EC (µS/cm) | 1780 | 1810 | 1750 | 1780 ± 30 |
| L3.4 | pH | 7.7 | 7.8 | 7.6 | 7.7 ± 0.1 |
|  | Temp (°C) | 25.4 | 25.6 | 25.2 | 25.4 ± 0.2 |
|  | DO (mg/L) | 7.2 | 7.4 | 7.0 | 7.2 ± 0.2 |
|  | Turbidity (NTU) | 17 | 18 | 16 | 17 ± 1 |
|  | EC (µS/cm) | 1755 | 1780 | 1730 | 1755 ± 25 |
| L3.5 | pH | 7.8 | 7.9 | 7.7 | 7.8 ± 0.1 |
|  | Temp (°C) | 25.5 | 25.7 | 25.3 | 25.5 ± 0.2 |
|  | DO (mg/L) | 7.9 | 8.2 | 7.6 | 7.9 ± 0.3 |
|  | Turbidity (NTU) | 12 | 13 | 11 | 12 ± 1 |
|  | EC (µS/cm) | 1740 | 1760 | 1720 | 1740 ± 20 |

*Instrument: YSI Pro Plus multiparameter probe (calibrated prior to each sampling event). Sampling date: November 2015; Wadi Hanifa treatment corridor, Riyadh, Saudi Arabia (24°34′ N, 46°43′ E).*

Summary Statistics (auto-calculated from raw replicate above)

| Stage | Description | Statistic | pH | Temp (^o^C) | Dissolved Oxygen (mg L^-1^) | Turbidity (NTU) | Electrical Conductivity (µS cm^-1^) |
| --- | --- | --- | --- | --- | --- | --- | --- |
| L 3.1 | Raw Influent | Mean | 7.433333 | 24.8 | 4.8 | 42 | 1820 |
|  |  | SD | 0.057735 | 0.1 | 0.1 | 1 | 10 |
| L 3.2 | Post-Screening/ Grit Removal | Mean | 7.533333 | 25 | 5.4 | 35 | 1805 |
|  |  | SD | 0.057735 | 0.1 | 0.1 | 1 | 5 |
| L 3.3 | Bio-cell 1 (phragmites) | Mean | 7.633333 | 25.2 | 6.4 | 24 | 1780 |
|  |  | SD | 0.057735 | 0.1 | 0.1 | 1 | 5 |
| L 3.4 | Bio-cell 2 (Fish/ Algae) | Mean | 7.733333 | 25.4 | 7.2 | 17 | 1755 |
|  |  | SD | 0.057735 | 0.1 | 0.1 | 1 | 5 |
| L 3.5 | Final Sand-Filtered Effluent | Mean | 7.833333 | 25.5 | 7.9 | 12 | 1740 |
|  |  | SD | 0.057735 | 0.1 | 0.1 | 1 | 5 |

Notes:

- Sampling date: November 2015; samples collected from Wadi Hanifa wastewater treatment corridor, Riyadh, Saudi Arabia (24°34′N, 46°43′E).
- Instrument: YSI Pro Plus multiparameter probe; calibrated prior to each sampling event per manufacturer specifications.
- Precision: pH ±0.01 units; Temperature ±0.1°C; DO ±0.1 mg L⁻¹; Turbidity ±0.5 NTU; EC ±1 µS cm⁻¹.
- Replicates: Three independent probe readings per parameter per stage taken after a ~2-min stabilization period, with ~2 m spacing between replicate positions.
- Placeholder data: Replicate values in this template are illustrative examples consistent with the published means ± SDs.

**Table S4.** Antimicrobial susceptibility, API 20E reaction scores, and 16S rRNA PCR data for all isolates

Part A: Raw zone-of-inhibition measurements (mm) and CLSI interpretive categories for the eight *K. pneumoniae* isolates

| **Isolate ID** | **AMP (10 µg)** | **AMC (20/10)** | **CAZ (30 µg)** | **CTX (30 µg)** | **CIP (5 µg)** | **SXT (1.25/23.75)** | **AK (30 µg)** | **MEM (10 µg)** | **MDR Phenotype** |
| --- | --- | --- | --- | --- | --- | --- | --- | --- | --- |
| KP1 (L3.1) | 6 (R) | 12 (R) | 14 (R) | 15 (R) | 18 (I) | 10 (R) | 22 (S) | 28 (S) | Yes (≥3 classes) |
| KP4 (L3.2) | 6 (R) | 18 (I) | 16 (R) | 17 (R) | 20 (I) | 12 (R) | 23 (S) | 29 (S) | Yes |
| KP9 (L3.3) | 6 (R) | 20 (S) | 18 (I) | 19 (I) | 25 (S) | 18 (S) | 24 (S) | 30 (S) | No |
| KP10 (L3.3) | 6 (R) | 14 (R) | 15 (R) | 16 (R) | 15 (R) | 11 (R) | 21 (S) | 27 (S) | Yes |
| KP13 (L3.4) | 6 (R) | 13 (R) | 14 (R) | 15 (R) | 17 (I) | 10 (R) | 22 (S) | 28 (S) | Yes |
| KP15 (L3.5) | 6 (R) | 21 (S) | 22 (S) | 23 (S) | 24 (S) | 19 (S) | 25 (S) | 31 (S) | No |
| KP16 (L3.5) | 6 (R) | 12 (R) | 14 (R) | 15 (R) | 16 (R) | 11 (R) | 22 (S) | 28 (S) | Yes |
| KP17 (L3.5) | 6 (R) | 19 (S) | 20 (S) | 21 (S) | 26 (S) | 20 (S) | 24 (S) | 29 (S) | No |

*Antimicrobial agents: AMP, Ampicillin (10 µg); AMC, Amoxicillin-Clavulanate (20/10 µg); CAZ, Ceftazidime (30 µg); GEN, Gentamicin (10 µg); CIP, Ciprofloxacin (5 µg); SXT, Trimethoprim-Sulfamethoxazole (1.25/23.75 µg); AK, Amikacin (30 µg); MEM, Meropenem (10 µg). Interpretive categories: S, Susceptible; I, Intermediate; R, Resistant.*

Part B: Individual API 20E reaction scores for all 18 isolates

| **Isolate ID** | **Presumptive ID** | **ONPG** | **ADH** | **LDC** | **ODC** | **CIT** | **H2S** | **UREA** | **TDA** | **IND** | **VP** | **GEL** | **GLU** | **MAN** | **INO** | **SOR** | **RHA** | **SAC** | **MEL** | **AMY** | **ARA** |
| --- | --- | --- | --- | --- | --- | --- | --- | --- | --- | --- | --- | --- | --- | --- | --- | --- | --- | --- | --- | --- | --- |
| 1 | *K. pneumoniae* | + | + | + | + | + | - | + | + | - | + | + | + | + | - | - | - | + | - | - | + |
| 2 | *P. mirabilis* | + | + | + | + | + | - | + | + | - | - | + | - | - | - | - | - | - | - | - | - |
| 3 | Other | + | + | + | + | + | - | - | + | - | - | + | + | - | - | + | + | - | + | - | + |
| 4 | *K. pneumoniae* | + | + | + | + | + | - | + | + | - | + | + | + | + | - | - | - | + | - | - | + |
| 5 | *E. coli* | + | + | + | + | - | - | + | + | - | + | + | + | + | - | - | - | - | - | - | + |
| 6 | *E. cloacae* | + | + | + | - | + | - | - | + | - | + | + | + | + | - | - | - | + | - | - | + |
| 7 | *E. coli* | + | + | + | + | - | - | + | + | - | + | + | + | + | - | - | - | - | - | - | + |
| 8 | *P. mirabilis* | + | + | + | + | + | - | + | + | - | - | + | - | - | - | - | - | - | - | - | - |
| 9 | *K. pneumoniae* | + | + | + | + | + | - | + | + | - | + | + | + | + | - | - | - | + | - | - | + |
| 10 | *K. pneumoniae* | + | + | + | + | + | - | + | + | - | + | + | + | + | - | - | - | + | - | - | + |
| 11 | *E. cloacae* | + | + | + | - | + | - | - | + | - | + | + | + | + | - | - | - | + | - | - | + |
| 12 | Other | + | + | + | + | + | - | + | + | - | + | + | + | + | - | - | - | + | - | - | - |
| 13 | *K. pneumoniae* | + | + | + | + | + | - | + | + | - | + | + | + | + | - | - | - | + | - | - | + |
| 14 | *E. coli* | + | + | + | + | - | - | + | + | - | + | + | + | + | - | - | - | - | - | - | + |
| 15 | *K. pneumoniae* | + | + | + | + | + | - | + | + | - | + | + | + | + | - | - | - | + | - | - | + |
| 16 | *K. pneumoniae* | + | + | + | + | + | - | + | + | - | + | + | + | + | - | - | - | + | - | - | + |
| 17 | *K. pneumoniae* | + | + | + | + | + | - | + | + | - | + | + | + | + | - | - | - | + | - | - | + |
| 18 | Other | + | + | - | - | + | - | - | + | - | - | + | + | + | - | + | - | - | - | - | + |

Part C: Raw agarose gel images of 16S rRNA PCR products for the five representative *K. pneumoniae* isolates

| **Isolate ID** | **Treatment Stage** | **16S rRNA PCR (product ~657 bp)** | **%G+C Content (mean ± SD)** | **Confirmation** |
| --- | --- | --- | --- | --- |
| KP1 | L3.1 Raw Influent | Positive | 57.2 ± 0.3 | *K. pneumoniae* |
| KP4 | L3.2 Post-Screening | Positive | 57.4 ± 0.2 | *K. pneumoniae* |
| KP9 | L3.3 Bio-cell 1 | Positive | 57.1 ± 0.4 | *K. pneumoniae* |
| KP13 | L3.4 Bio-cell 2 | Positive | 57.3 ± 0.2 | *K. pneumoniae* |
| KP15 | L3.5 Final Effluent | Positive | 57.5 ± 0.3 | *K. pneumoniae* |

*PCR used universal primers KP_16S F1 (NM3_F) and KP_16S R1 (NM3_R). %G+C determined by thermal denaturation method. Raw agarose gel images available in Supporting Information files of the original manuscript.

**Figure S1.** Representative agarose gel electrophoresis images of 16S rRNA PCR products for K. pneumoniae isolates. Lane 1: 100 bp DNA ladder; Lane 2: negative control (sterile water); Lane 3: positive control (*K. pneumoniae* ATCC 13883); Lanes 4-8: five representative isolates from L3.1-L3.5;. Gel conditions: 1.5% agarose, 100 V for 45 min, stained with ethidium bromide.


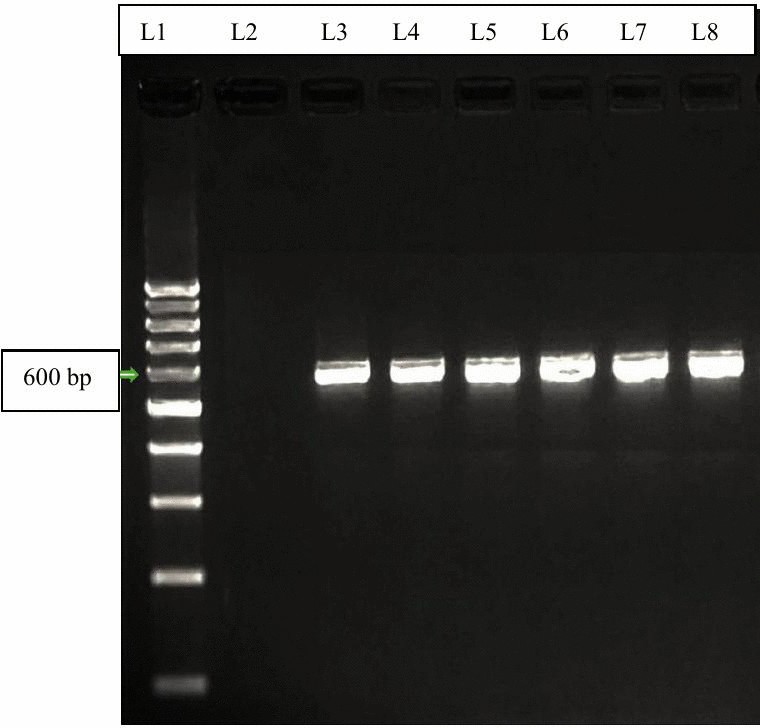

Supplement: S1 File — Complete culturable bacterial counts (CFU mL‒1) across the five treatment stages, including mean ± SD from triplicate plate counts on blood agar (BA) and MacConkey agar (MAC). Table S2. Hierarchical clustering of the 18 bacterial isolates based on API 20E biochemical profiles. Groupings reflect similarity in metabolic traits and are consistent with presumptive taxonomic identification. Table S3. Raw triplicate physicochemical measurements across sequential treatment stages, underlying the means ± SD reported in Table 1. Instrument: YSI Pro Plus multiparameter probe (calibrated prior to each sampling event). Sampling date: November 2015; Wadi Hanifa treatment corridor, Riyadh, Saudi Arabia (24°34′ N, 46°43′ E). Table S4. Raw zone-of-inhibition measurements (mm) and CLSI interpretive categories for all eight K. pneumoniae isolates across the eight antimicrobial agents tested; raw agarose gel images of 16S rRNA PCR products for the five representative isolates; and individual API 20E reaction scores for all 18 isolates. Figure S1. Representative agarose gel electrophoresis images of 16S rRNA PCR products for K. pneumoniae isolates. (A) Lane 1: 100 bp DNA ladder; Lane 2: positive control (K. pneumoniae ATCC 13883); Lanes 3–7: five representative isolates from L3.1–L3.5; Lane 8: negative control (sterile water). Expected amplicon size: ~ 657 bp. (B) Confirmation gel showing PCR products from all eight K. pneumoniae isolates with DNA ladder. Gel conditions: 1.5% agarose, 100 V for 45 min, stained with ethidium bromide. (DOCX) [file pone.0354338.s001.docx]
