# Supplementary material for: Persistence of viable opportunistic pathogens in a multi-stage natural wastewater treatment system
Source: PLoS One. 2026 Jul 27;21(7):e0354338. doi: 10.1371/journal.pone.0354338 (PMC13405070; doi:10.1371/journal.pone.0354338)
Supplement: Attachment — Submitted filename: Response to Reviewers.docx [file pone.0354338.s003.docx]

**Reviewer #1 (Mohamed Azab El-Liethy) – Major Comments**

**R1-Major 1: Justification of API system**

*The authors relied on the API system. More recent automated systems such as Biolog and VITEK are now widely used. The authors should justify the use of the API system in the Introduction.*

Response: We thank the reviewer for this valid point. We have added the following justification in the Introduction: 'The API system (bioMérieux) was selected for biochemical identification because it provides a standardized, cost-effective, and well-validated approach for environmental isolate identification, with an established database widely used in both clinical and environmental microbiology. This choice was also consistent with the resources available at the time of sample processing (2015).' We acknowledge that automated platforms such as VITEK 2 or MALDI-TOF MS would provide faster and potentially more accurate identification and should be considered in future studies.

**R1-Major 2: Absence of E. coli in downstream samples**

*It is unclear why E. coli was not detected in downstream samples while other organisms were still present. The use of selective media specifically targeting E. coli might have yielded different results.*

Response: This is an important point. We have expanded the discussion to address this observation explicitly. In the revised manuscript, we identify three plausible explanations: (i) the use of non-selective BA and MAC agar rather than chromogenic media (e.g., CHROMagar Orientation or Brilliance *E. coli*/Coliform agar) may have resulted in underestimation of E. coli in downstream samples; (ii) *E. coli* is known to be more susceptible to environmental stressors (elevated DO, reduced organic matter, UV) than non-enteric facultative Gram-negatives; and (iii) the very low abundance at downstream stages may have fallen below the detection limit of our plating approach. We have added this discussion and acknowledged the use of non-selective media as a methodological limitation.

**R1-Major 3: Limited number of isolates**

*The number of isolates is too limited to support reliable conclusions. The total number of isolates and the number from each stage must be clearly reported in Materials and Methods.*

Response: We agree that this is a significant limitation. We have (i) clearly stated in the Methods (Bacterial isolation and enumeration) that a total of 18 morphologically distinct colonies were selected across all five treatment stages (approximately 3–4 isolates per stage), and have described the morphotype-based selection criteria; (ii) explicitly framed the study as exploratory and preliminary throughout the manuscript, including in the Abstract, Results and Discussion, and Conclusions; and (iii) added a statement in the Conclusions recommending larger, multi-season studies with expanded isolate collections to validate these findings.

**R1-Major 4: Results and Discussion depth**

*The Results and Discussion section lacks depth and clarity and requires significant revision.*

Response: The Results and Discussion section has been substantially revised. Key improvements include: (i) expanded interpretation of physicochemical data (pH, temperature, EC) and their relevance to microbial communities; (ii) more detailed mechanistic discussion of *K. pneumoniae* persistence; (iii) expanded comparison with recent studies from similar climates and systems; (iv) cautious language throughout to reflect the small sample size; and (v) reduced redundancy between tables and text.

**R1-Specific 1: Italics for 'viable'**

*Line 23: write the word 'viable' in non-italic throughout the manuscript.*

Response: Corrected throughout the manuscript. The word 'viable' is not a microorganism name and has been changed to roman text.

**R1-Specific 2: Sentence on Wadi Hanifa statistics**

*Line 55: transfer this sentence to the Materials and Methods section.*

Response: The sentence describing the volume received by the Wadi Hanifa system (300,000 m³ day⁻¹) has been moved to the Study area and sampling design subsection of Methods. We have also added that the system serves approximately 7 million people in the greater Riyadh metropolitan area.

**R1-Specific 3: Merge study area subsections and remove Figure 1**

*Line 85: merge the two parts about Study area and sampling design into one and delete Fig 1 as Figure 2 is sufficient.*

Response: The two subsections (Study site description and Sampling locations) have been merged into a single section titled 'Study area and sampling design.' Figure 1 & Figure 2 has been removed (also addressed under Journal Requirement 4 regarding copyright).

**R1-Specific 4: Sampling date and population served**

*Line 90: The 2015 sampling date needs justification for current relevance. Also, the number of people served by the plant should be mentioned.*

Response: We have added an explanation that the November 2015 sampling date represents stable dry-season operating conditions and that the treatment configuration has remained consistent. We have also added a note on the population served (~7 million, greater Riyadh area). We acknowledge that 10-year-old data are a limitation and have framed the study accordingly.

**R1-Specific 5: Sampling location description and effluent use**

*Line 100: Rewrite sampling location descriptions as complete sentences. Clarify whether a final disinfection step is applied and how the final effluent is used.*

Response: The bullet-point list of sampling locations has been replaced with full sentences in the revised manuscript. We have added that no terminal chlorination step is applied and that the final effluent is used for landscape irrigation and green belt maintenance in Riyadh.

**R1-Specific 6: References for sample collection procedure**

*Line 118: Provide appropriate references for sample collection and transportation methods.*

Response: A reference to the Standard Methods for the Examination of Water and Wastewater (APHA, 2023; reference 32) has been added to the sample collection paragraph.

**R1-Specific 7: COD determination**

*Line 124: COD is an important parameter. If not measured, justify its omission.*

Response: COD was not measured in this study. We have added the following statement in the Physicochemical analysis section: 'Chemical oxygen demand (COD) was not measured in this study; determination of COD would have required a separate sampling and analytical setup that was not available at the time of field collection. Future studies should include COD as a standard organic loading parameter.'

**R1-Specific 8: Antibiotic names in Methods**

*Line 151: the names of antibiotics tested should be mentioned in this section.*

Response: The full list of eight antimicrobial agents tested (with drug class and disk concentration) has been added to the Antimicrobial susceptibility testing subsection: ampicillin (10 µg), amoxicillin-clavulanate (20/10 µg), ceftazidime (30 µg), gentamicin (10 µg), ciprofloxacin (5 µg), trimethoprim-sulfamethoxazole (1.25/23.75 µg), amikacin (30 µg), and meropenem (10 µg).

**R1-Specific 9: Discussion of pH, temperature, and EC**

*Line 179: The authors neglected to discuss pH, temperature, and EC.*

Response: A dedicated discussion of all physicochemical parameters has been added. We now address pH (modest increase from 7.4 to 7.8, consistent with photosynthetic activity in bio-cells), temperature (stable at 24.8–25.5°C, reflecting dry-season conditions), and EC (minimal variation, indicating limited alteration of dissolved salts). We discuss the implications of the stability of these parameters for the observed microbial community changes.

**R1-Specific 10: Regulatory limits for wastewater reuse**

*Line 198: The authors should clarify the regulatory limits for wastewater reuse and justify the parameters used to assess suitability for irrigation.*

Response: We have revised this paragraph to clarify that the WHO ≥5-log reduction target refers to pathogen-specific reductions (e.g., for helminth eggs, E. coli, and rotavirus) rather than total culturable bacterial counts, and that the comparison is provided for context only. We note that most national and international guidelines are based primarily on *E. coli*/fecal coliforms and discuss this in the context of the limitations of indicator-based monitoring highlighted by our findings.

**R1-Specific 11: API confidence calculation**

*Line 219: How is API confidence % calculated? This should be clarified in Methods.*

Response: We have added an explanation in the Phenotypic, biochemical, and molecular characterization subsection: 'Identification was accepted at ≥80% confidence using the apiweb™ database; the percentage confidence reflects the probability of the given identification based on the biochemical reaction profile.' We have also added a footnote to Table 3 to this effect.

**Reviewer #2 – Major Comments**

**R2-Major 1: Sampling design clarity**

*The sampling design and experimental workflow need more detail: sampling frequency, number of replicates, distribution of sampling points.*

Response: We have clarified in the Methods that: (i) sampling was conducted on a single occasion (November 2015), representing a single time point; (ii) triplicate technical replicates were collected at each of the five sampling locations; (iii) a total of 18 morphologically distinct isolates were selected across all stages. We acknowledge the single time-point as a study limitation and recommend repeated sampling in future studies.

**R2-Major 2: Molecular confirmation**

*Identification relies mainly on culture-based methods. Including molecular confirmation such as 16S rRNA sequencing would increase reliability.*

Response: We have clarified in the Methods that representative *K. pneumoniae* isolates from each treatment stage (n = 5) were confirmed by partial 16S rRNA gene sequencing (∼1,400 bp) with ≥99% identity to reference sequences, as was described in the original manuscript.

**R2-Major 3: Antimicrobial resistance analysis**

*Resistance patterns are not discussed in sufficient detail. Clearer presentation and interpretation with public health implications would strengthen the manuscript.*

Response: The antimicrobial resistance section has been expanded. We now discuss: (i) the significance of universal ampicillin resistance as intrinsic to *K. pneumoniae*; (ii) the elevated resistance to ceftazidime and amoxicillin-clavulanate as suggestive of possible ESBL activity; (iii) the retention of susceptibility to amikacin and meropenem as reassuring for clinical management; and (iv) the public health implications of viable MDR *K. pneumoniae* in irrigation effluent, while noting that quantitative risk assessment was not performed.

**R2-Major 4: Data presentation and statistics**

*Results would benefit from clearer presentation and more detailed statistical analysis. Specify statistical tests, confidence levels, and rationale for correlations.*

Response: All statistical tests are now explicitly stated in the Methods and referenced in the Results. Justification for the choice of Kendall’s τ, linear regression, and Cochran–Armitage tests given the small sample size has been added. Confidence levels and p-values are reported consistently throughout.

**R2-Major 5: Depth of discussion**

*The discussion could be strengthened by comparing findings with recent studies on pathogen persistence in natural wastewater treatment systems.*

Response: The discussion has been substantially expanded with comparisons to recent studies from similar arid-region and nature-based treatment contexts, including studies from Saudi Arabia (reference 30) and other arid environments. The implications for QMRA and wastewater reuse policy in water-scarce regions have been discussed more thoroughly.

**R2-Minor Comments**

*Language revision, consistent italics for bacterial names, abbreviations at first use, clearer figure/table legends, minor grammatical corrections.*

Response: All these points have been addressed: (i) the manuscript has been carefully proofread; (ii) all bacterial names are italicized consistently; (iii) all abbreviations are defined at first use (FIB, DO, EC, NTU, ARG, DNA, QMRA, MDR, CFU, PBS, BA, MAC, CLSI); (iv) table captions have been improved for clarity; and (v) grammatical corrections have been made throughout.

**Reviewer #3 – Major Issues**

**R3-Major 1: Title page formatting**

*The label 'Manuscript Title:' should be removed. ORCID identifiers should be in URL format. Phone number should be removed.*

Response: All three issues have been corrected: the 'Manuscript Title:' label has been removed; ORCID identifiers are now provided in URL format (https://orcid.org/XXXX-XXXX-XXXX-XXXX); and the phone number has been removed from the corresponding author block.

**R3-Major 2: Abstract revisions**

*The study objective should be stated more directly; culture-based identification described carefully; findings described as exploratory; percentages include actual numbers; correlation interpreted cautiously; MDR statement softened; indicator monitoring limitation described as system-specific; add 'antimicrobial resistance' as keyword.*

Response: The Abstract has been revised to address all these points: (i) the study objective is stated directly at the outset; (ii) culture-based identification is qualified as providing presumptive identification; (iii) the findings are framed as exploratory; (iv) percentages now include absolute counts in parentheses (e.g., 44.4%, 8/18; 62.5%, 5/8); (v) the Kendall’s τ correlation is accompanied by a caution regarding small sample size; (vi) the MDR statement now reads 'under the conditions examined'; (vii) indicator monitoring limitations are described as system-specific; and (viii) 'antimicrobial resistance' has been added to the keywords.

**R3-Major 3: Introduction improvements**

*Research gap should be stated more explicitly and earlier. Rationale for K. pneumoniae focus should include environmental persistence and irrigation exposure. More regional/local studies should be cited. Introduction should end with hypotheses.*

Response: The Introduction has been revised: (i) the research gap (limited culture-based viability data for opportunistic pathogens in nature-based treatment systems) is now stated explicitly in the second paragraph; (ii) the rationale for focusing on K. pneumoniae is strengthened with reference to its capsule-mediated environmental persistence and relevance to irrigation-related exposure; (iii) an additional regional reference (Booq et al., 2022, reference 30) from Saudi Arabia is cited; and (iv) the Introduction ends with an explicit hypothesis statement.

**R3-Major 4: Methods improvements**

*Explain November 2015 sampling date and seasonal variation. Clarify isolate selection criteria and total number. Clarify technical vs. biological replicates. Acknowledge selective bias of BA/MAC. Specify how many isolates confirmed by 16S. Include antibiotic list. Justify statistics for small sample size. Add detail on calibration and contamination control during sampling.*

Response: All these points have been addressed in the revised Methods: (i) sampling in November is noted as representing stable dry-season conditions, with seasonal variation acknowledged as a limitation; (ii) the total of 18 isolates and the per-stage approximate count are now stated, along with the morphotype-based selection criteria; (iii) triplicates are clarified as technical replicates collected at the same time point and location; (iv) the culture-based bias toward aerobic and facultatively anaerobic heterotrophs is acknowledged as a limitation; (v) it is clarified that 5 representative *K. pneumoniae* isolates (one per stage) were confirmed by 16S rRNA sequencing; (vi) the full antibiotic list with disk concentrations is now provided; (vii) justification for the statistical approaches is provided in the Statistical analysis section; and (viii) detail on probe calibration, autoclaved sample containers, and field blanks has been added to the Quality assurance subsection.

**R3-Major 5: Results and Discussion improvements**

*Some subsections could be merged; WHO comparison should specify pathogen removal; redundancy between tables and text; system-specific conclusions; percentages with absolute numbers; cautious correlation interpretation; moderate statements about global AMR reservoirs; acknowledge other bacterial reduction mechanisms; public health implications should not be overstated; acknowledge limitations; expand comparison with previous studies.*

Response: All these points have been addressed: (i) the 'Bacterial diversity' and 'Correlations' subsections have been merged; (ii) the WHO comparison is clarified as referring to pathogen-specific targets; (iii) text redundancy with tables has been reduced; (iv) indicator monitoring conclusions are qualified as system-specific; (v) all percentages now include absolute counts; (vi) all correlation statements include a caution regarding small sample size; (vii) global AMR reservoir statements are moderated; (viii) other possible mechanisms (UV, predation, competition) are mentioned; (ix) public health implications are appropriately qualified; (x) limitations are explicitly acknowledged; and (xi) comparison with previous studies has been expanded.

**R3-Major 6: Conclusions**

*The conclusion is somewhat overstated. The statement about 'not eliminating' pathogens should be softened. Suggestions for monitoring/treatment steps should note practical limitations. The findings should be stated as preliminary, with larger long-term studies needed.*

Response: The Conclusions section has been substantially revised. The key change is that the findings are now explicitly described as preliminary, based on a single sampling event with a limited number of isolates (n = 18). The statement about pathogen non-elimination has been qualified as 'under the conditions examined.' The recommendation for terminal disinfection is accompanied by an acknowledgement that practical and economic constraints must be considered. A call for larger, multi-season studies is included.

**R3-Major 7: Data availability**

*The manuscript does not fully meet PLOS data availability requirements. Raw microbiological counts, isolate-level information, and detailed AMR results should be made fully accessible.*

Response: All underlying data are contained within the manuscript tables and Supporting Information files. Table 2 provides the full bacterial count data (mean ± SD from triplicate plate counts). Table 3 provides isolate-level identification data for all 18 isolates. Table 4 provides the complete antimicrobial susceptibility results for all 8 *K. pneumoniae* isolates. Table S1 provides the complete CFU data and Table S2 the hierarchical clustering results. The Data Availability Statement has been retained as: 'All data generated or analyzed during this study are included in this published article and its Supporting Information files.'

**R3-Minor Issues**

*Grammatical/typographical errors; consistent terminology (viable vs. culturable); tables and figures formatting; duplicate references; abbreviations at first use; consistent statistical reporting; reduce repetition.*

Response: All minor issues have been addressed: (i) the manuscript has been thoroughly proofread; (ii) terminology is now consistent throughout—we use 'culturable' when referring to organisms detected by culture and 'viable (culturable)' at first use to acknowledge this equivalence; (iii) tables have improved formatting and captions; (iv) duplicate references have been removed and the reference list renumbered; (v) all abbreviations are defined at first use; (vi) statistical reporting (p-values to three decimal places, consistent decimal places in tables) is now uniform; and (vii) repetition between Results and Discussion has been reduced.

**R3-Author Contributions**

*You might add that all authors approved the final version.*

Response: Added. The Author Contributions section now ends with: 'All authors have reviewed and approved the final version of the manuscript.'

**R3-References**

*Check for duplicates (16/34, 17/35, 29/37, 33/36) and ensure correct formatting.*

Response: Duplicate references have been identified and removed. References 34, 35, 36, and 37 in the original manuscript duplicated references 16, 17, 29, and 33, respectively. The duplicate entries have been removed and the reference list renumbered accordingly. All in-text citations have been updated.
