# Supplementary material for: Persistence of viable opportunistic pathogens in a multi-stage natural wastewater treatment system
Source: PLoS One. 2026 Jul 27;21(7):e0354338. doi: 10.1371/journal.pone.0354338 (PMC13405070; doi:10.1371/journal.pone.0354338)
Supplement: Attachment — Submitted filename: Response_to_Reviewers.docx [file pone.0354338.s004.docx]

**RESPONSE TO REVIEWERS**

Manuscript PONE-D-26-01733R1

*"Persistence of Viable Opportunistic Pathogens in a Multi-Stage Natural Wastewater Treatment System"*

**Dr. Muhammad Shahid, PhD**

Academic Editor, PLOS ONE

Date: **25 May 2026**

Re: Manuscript **PONE-D-26-01733R1** — Second Revision

**Response to Reviewers**

**Note:** Reviewer #1 and Reviewer #2 have confirmed that all their previous comments were fully addressed. The responses below address all comments raised by Reviewer #3.

**Reviewer #1 & #2**

**Status:** All comments addressed and accepted.

**Reviewer #3 — Point-by-Point Response**

**MAJOR ISSUES**

**Comment 1 — Figure numbering inconsistency**

*"The Methods section still refers to 'Fig 1,' although the manuscript indicates that Figure 1 was removed due to copyright concerns. Figure references should be checked carefully and updated consistently throughout the manuscript."*

**Response:** We have removed all residual references to "Fig 1" throughout the manuscript and verified that no figure citations remain orphaned. The Methods section now describes the sampling configuration entirely in text without reference to a figure. All Table references (Tables 1–8 and Tables S1–S4) have been re-checked for consistency.

**Comment 2 — Temporal limitation of the dataset (November 2015 samples)**

*"Adding a brief statement in the limitations section explaining the continuing value of these data would strengthen the manuscript further."*

**Response:** We agree. We have expanded the limitations statement in both the Methods (Study Area subsection) and the Conclusions to explicitly justify the continuing value of the 2015 dataset. The added text reads:

*"Although the samples analyzed in this study were collected in November 2015, the operational configuration, hydraulic residence time (4–6 d), vegetation regime, and effluent-reuse pattern of the Wadi Hanifa treatment corridor have remained substantively unchanged since that time, as confirmed through recent operational reports of the Riyadh municipal water authority. The dataset therefore retains relevance as a baseline characterization of viable opportunistic pathogen persistence in this system. Furthermore, culture-based viability data of this granularity remain scarce for arid-region nature-based treatment systems, and the dataset addresses a persistent gap in the literature regardless of collection year."*

**Comment 3 — Interpretation of statistical findings**

*"Some statements could still be softened slightly... 'declined by 50%' could be rephrased as 'declined by approximately 50%'."*

**Response:** We have systematically softened statistical statements throughout. Key changes: Abstract: "a 50% reduction" → "an approximately 50% reduction in observed isolate richness". Results (Diversity): "Isolate richness declined by 50%..." → "Observed isolate richness declined by approximately 50%...(Cochran–Armitage exact test, p = 0.022; n = 5 stages, exploratory)". A clarifying sentence was added to the Statistical Analysis section:

*"All statistical analyses presented here are exploratory and intended to identify trends consistent with biological mechanisms, rather than to provide definitive inferential conclusions."*

**Comment 4 — Public health interpretation (AMR reservoir/dissemination claims)**

*"A few statements regarding wastewater systems as reservoirs or dissemination pathways for antimicrobial resistance should still be moderated slightly."*

**Response:** We have moderated these statements in three locations (Abstract, AMR section, Implications). The revised phrasing now reads:

*"...consistent with recent reports suggesting that wastewater systems may act as environmental compartments where antimicrobial-resistant organisms can persist; however, given the limited number of isolates (n = 8) and the absence of molecular resistance gene characterization in this study, broader claims regarding dissemination cannot be made and would require dedicated genomic and longitudinal investigation."*

**Comment 5 — Supporting Information and raw data availability**

*"The authors should clarify whether the raw microbiological counts, physicochemical measurements, antimicrobial susceptibility data, and sequencing results are included within the Supporting Information files or deposited in a public repository."*

**Response:** We have substantially revised the Data Availability Statement and added a new Supporting Information table (Table S4) containing the complete raw antimicrobial susceptibility zone-of-inhibition measurements for all eight K. pneumoniae isolates. The updated Data Availability Statement is included in full in the revised manuscript. GenBank accession numbers will be confirmed at the proof stage; a placeholder is included. We have also added explicit in-text citations to Supporting Information files at each relevant point in the Results.

**MINOR ISSUES**

**Comment 6 — Proofreading, formatting, hyphenation**

*"A careful proofreading is still recommended to correct a few remaining formatting inconsistencies and typographical errors. Hyphenation and formatting should be standardized throughout the manuscript."*

**Response:** The manuscript has been thoroughly proofread. Hyphenation is now standardized throughout: "multi-stage," "sand-filtered," "culture-based," "nature-based," and "fecal-indicator." Residual formatting inconsistencies (spacing, decimal alignment, italicization of taxonomic names) have been corrected.

**Comment 7 — Terminology consistency ('viable,' 'culturable,' 'culture-based')**

*"Terminology should remain consistent throughout the manuscript, particularly regarding 'viable,' 'culturable,' and 'culture-based' bacteria."*

**Response:** We have systematically standardized terminology throughout. A clarifying Terminology Note has been added to the Methods section defining each term: 'culturable' = bacteria recoverable under specific media/incubation conditions; 'viable' = synonym only insofar as culturability implies viability (with VBNC caveat); 'culture-based' = methodological framework.

**Comment 8 — Statistical and decimal formatting**

*"Statistical reporting and decimal formatting should be checked for consistency."*

**Response:** All p-values are now reported to three decimal places (e.g., p = 0.022, p = 0.038, p = 0.062). Correlation coefficients are reported to two decimal places. Means ± SD use consistent significant-figure conventions throughout.

**Comment 9 — Repetition between Results and Discussion**

*"Some repetition between the Results and Discussion sections could still be reduced slightly."*

**Response:** The Implications subsection has been condensed; it now cross-references the earlier sub-section rather than restating findings about the E. coli/K. pneumoniae contrast. Specific restatements of bacterial counts and diversity metrics have been removed from the Implications section.

**Comment 10 — WHO comparison clarification**

*"The WHO comparison is now better qualified, but the manuscript should continue clarifying that WHO benchmarks refer to pathogen-specific reductions rather than total culturable bacterial counts."*

**Response:** We have strengthened the clarification:

*"It is important to note that WHO ≥5-log reduction targets refer to pathogen-specific reductions (e.g., for rotavirus, Cryptosporidium, and bacterial pathogens at the unit-process level), not to total culturable heterotrophic bacterial counts. The comparison here is provided solely for contextual scale; total CFU reduction is not a direct equivalent of pathogen-specific log-removal credits and should not be interpreted as such."*

**Comment 11 — Additional references in Results and Discussion**

*"The Results and Discussion sections would benefit from additional references to strengthen comparisons with previous studies."*

**Response:** We have added six relevant references (#35–40) to strengthen comparisons on pathogen persistence, AMR in wastewater, and limitations of fecal indicators. Full citations are listed in the revised reference list.

**Comment 12 — Clustering table relevance in main text**

*"The clustering table is informative, but a brief explanation of its relevance to pathogen persistence within the main text would improve clarity."*

**Response:** We added the following sentence to the Results section (Shifts in viable bacterial composition subsection):

*"The hierarchical clustering of biochemical profiles (Supporting Table S2) showed that all eight K. pneumoniae isolates—recovered across the full treatment train—grouped within a tight metabolic cluster distinct from E. coli, Enterobacter, and Proteus isolates, indicating phenotypic coherence of the persistent K. pneumoniae population and consistency of identification across stages."*

**Comment 13 — Reference list final check**

*"The duplicate reference issue appears to have been addressed, although the final reference list should still be checked carefully before submission."*

**Response:** The reference list has been re-verified in full. No duplicates remain. All 40 references follow PLOS ONE Vancouver style consistently. The six new references (#35–40) have been integrated and numbered sequentially.

**Summary of All Revisions**

| **#** | **Reviewer Concern** | **Action Taken** |
| --- | --- | --- |
| 1 | Figure 1 references | All removed; manuscript is fully figure-free; all table references re-checked |
| 2 | 2015 dataset relevance | New justification added to Methods (Study Area) and Conclusions |
| 3 | Statistical phrasing too strong | All claims softened; exploratory framing added throughout |
| 4 | AMR reservoir/dissemination overreach | Moderated in Abstract, AMR section, and Implications (3 locations) |
| 5 | Raw data availability unclear | New Supporting Table S4; expanded Data Availability Statement; GenBank deposition noted |
| 6 | Proofreading / hyphenation | Standardized throughout: multi-stage, sand-filtered, culture-based, nature-based |
| 7 | Terminology consistency | New 'Terminology Note' added in Methods section |
| 8 | Decimal/statistical formatting | Uniform 3 d.p. p-values, 2 d.p. τ values, consistent SD conventions |
| 9 | Redundancy Results/Discussion | Implications section condensed; cross-references used instead of repetition |
| 10 | WHO benchmark clarification | Explicit clarifying statement added |
| 11 | Additional references | 6 new references (#35–40) integrated at appropriate locations |
| 12 | Clustering table relevance | Explanatory sentence added in Results section |
| 13 | Reference list duplicates | Re-verified — no duplicates remain |
