# Supplementary material for: Persistence of viable opportunistic pathogens in a multi-stage natural wastewater treatment system
Source: PLoS One. 2026 Jul 27;21(7):e0354338. doi: 10.1371/journal.pone.0354338 (PMC13405070; doi:10.1371/journal.pone.0354338)
Supplement: Attachment — Submitted filename: Response_to_Reviewers_auresp_3.docx [file pone.0354338.s005.docx]

**Response to Reviewers' Comments**

**Manuscript:** PONE-D-26-01733R2
**Title:** "Persistence of Viable Opportunistic Pathogens in a Multi-Stage Natural Wastewater Treatment System"

**Response to Reviewer #3:**

We thank Reviewer #3 for the careful and constructive evaluation of our revised manuscript. We are encouraged that the reviewer found the manuscript "improved considerably" and recommended acceptance after minor revision. We have addressed each of the remaining comments in detail below.

**Review Summary**

We appreciate the reviewer's overall positive assessment that "the authors have made a substantial effort to address the comments raised during the previous round of review" and that "the revised manuscript has improved considerably in terms of clarity, scientific interpretation, and transparency."

We are grateful for the recognition that our revisions have "strengthened the overall quality of the manuscript" and that "the additional explanations, revised discussion, supporting information, and updated references have improved both the readability and scientific value of the study."

**Responses to Specific Comments**

**Terminology (Comment 1)**

**Reviewer Comment:** "I appreciate the addition of the terminology note. However, the statement indicating that 'viable' is used as a synonym for 'culturable' should be reconsidered. From a microbiological perspective, recovery by culture provides evidence of viability under the specific experimental conditions, but the two terms are not synonymous because viable-but-non-culturable (VBNC) bacteria remain viable despite not being recoverable by conventional culture methods. A slight revision of this wording would improve the scientific accuracy of the manuscript."

**Response:** We agree with the reviewer's important distinction and have revised the Terminology Note to more accurately reflect the relationship between viability and culturability. The revised note now reads:

**Terminology Note.** Throughout this manuscript, "culturable" refers to bacteria recoverable under the specific media and incubation conditions used in this study. "Viable" is used in the conventional sense of having metabolic activity and the potential for growth; however, culturability is considered a proxy for viability rather than a perfect synonym, as the viable-but-non-culturable (VBNC) state is well documented for many bacterial taxa, including Klebsiella pneumoniae, and is not captured by the culture-based approach used here. "Culture-based" describes the overall methodological framework, and findings should be interpreted with the understanding that VBNC cells are not enumerated.

**[Location:** Materials and Methods, Study Area and Sampling Design subsection]

**Table Footnotes (Comment 2)**

**Reviewer Comment:** "Although the tables are well organized and their titles are appropriate, I recommend adding footnotes below each table to define all abbreviations used. For example, abbreviations such as n (sample size), CFU (colony-forming units), PA (Pseudomonas aeruginosa), % (percentage), and any other abbreviations should be explained beneath the corresponding table. This will make each table self-contained and easier for readers to interpret without referring back to the main text."

**Response:** We have added comprehensive footnotes to all tables in the manuscript and Supporting Information to define all abbreviations used. The footnotes have been added as follows:

**Table 1 (Physicochemical parameters):**
*Footnote:* Values are mean ± standard deviation from triplicate replicate measurements at each sampling location (n = 3). DO = dissolved oxygen; EC = electrical conductivity; NTU = nephelometric turbidity units; Temp = temperature; °C = degrees Celsius; mg/L = milligrams per liter; μS/cm = microsiemens per centimeter.

**Table 2 (Culturable bacterial counts):**
*Footnote:* Values are mean ± standard deviation from triplicate plate counts (n = 3 plates per dilution). BA = blood agar; MAC = MacConkey agar; CFU = colony-forming units; mL = milliliter. The highest dilution yielding countable colonies was used for enumeration in each case.

**Table 3 (Bacterial isolates identification):**
*Footnote:* Presumptive identification based on API 20E biochemical profiles (bioMérieux, Marcy-l'Étoile, France). API codes are 7-digit numerical profiles; identification confidence based on manufacturer's database (≥85% considered acceptable). n = number of isolates; % = percentage of total isolates characterized; L3.1-L3.5 = sampling locations along treatment train (influent to final effluent). Isolate IDs correspond to morphologically distinct colonies selected from blood agar and MacConkey agar plates.

**Table 4 (Antimicrobial susceptibility patterns):**
*Footnote:* Antimicrobial susceptibility testing performed by Kirby-Bauer disk diffusion on Mueller-Hinton agar following CLSI M100 (33rd edition, 2023) guidelines. n = 8 K. pneumoniae isolates recovered across all treatment stages. MDR = multidrug resistance, defined as resistance to ≥3 antimicrobial classes. Percentages are based on n = 8 isolates. CLSI interpretive criteria: S = susceptible, I = intermediate, R = resistant. All isolates were tested in triplicate (raw zone-of-inhibition measurements provided in Supporting Table S4).

**Table 5 (Bacterial diversity metrics):**
*Footnote:* S = observed isolate richness (number of distinct morphotypes isolated per 500 mL sample). H' = Shannon-Wiener diversity index. 1-D = Simpson's diversity index. J' = Pielou's evenness (H'/ln S). Values are based on 18 morphologically distinct isolates characterized across five treatment stages. L3.1-L3.5 = sequential treatment stages from influent to final effluent.

**Table 6 (Kendall's τ correlations):**
*Footnote:* τ = Kendall's rank correlation coefficient; p = p-value (two-tailed). n = 5 treatment stages. *Significant at α = 0.05. DO = dissolved oxygen; EC = electrical conductivity. All p-values are reported as exploratory given the small sample size. Interpretations are qualitative and intended to identify trends consistent with biological mechanisms.

**Table 7 (Molecular characterization of representative K. pneumoniae isolates):**
*Footnote:* %G+C = guanine-plus-cytosine content determined by thermal denaturation method (Marmur and Doty, 1962). Values are mean ± standard deviation from triplicate thermal denaturation measurements. bp = base pairs. PCR = polymerase chain reaction. All five isolates confirmed as consistent with Klebsiella pneumoniae based on %G+C content (expected range: 57-58%) and 16S rRNA PCR amplicon size (expected size: ~657 bp). See Supporting Table S4 for raw agarose gel images.

**[Location:** Beneath each table in the main manuscript]

**Reference Formatting (Comment 3)**

**Reviewer Comment:** "The reference list has been improved substantially. However, before final acceptance, I recommend performing one final review to ensure that all references fully comply with the PLOS ONE formatting guidelines. In particular, the authors should verify the formatting of author lists, the appropriate use of et al., journal abbreviations, article numbers, DOI formatting (where applicable), and overall consistency across the reference list."

**Response:** We have performed a final comprehensive review of the reference list to ensure full compliance with PLOS ONE formatting guidelines. Specific checks conducted:

1. **Author lists:** Verified that for references with ≤6 authors, all authors are listed; for references with >6 authors, the first 6 are listed followed by "et al." This has been applied consistently.
2. **Journal abbreviations:** Standard ISO abbreviations have been used for all journals (e.g., Water Res, Sci Total Environ, PLoS One).
3. **Article numbers:** Where provided, article numbers (e.g., e0309409) are included and formatted correctly.
4. **DOI formatting:** DOIs have been added where available and formatted as hyperlinks.
5. **Reference order:** All references are numbered sequentially in the order of first citation in the text and are listed in the same order in the reference list.
6. **Consistency:** All references now follow a uniform format with respect to author names, article titles, journal names, volume, issue, pages, and year.

The reference list has been re-verified, and no duplicates remain. All 40 references are now fully compliant with PLOS ONE Vancouver style.

**[Location:** Reference list, pages 26-28]

**Supporting Figures (Comment 4)**

**Reviewer Comment:** "If available, I encourage the authors to include representative figures supporting the molecular findings, such as agarose gel electrophoresis images of PCR products or other relevant laboratory images, either in the main manuscript or as Supporting Information. Although this is not essential for acceptance, such figures would further enhance the transparency of the study and provide additional support for the reported molecular results."

**Response:** We agree with the reviewer that including representative molecular figures enhances transparency. We have prepared Supporting Figure S1 containing:

1. **Representative agarose gel electrophoresis image** showing 16S rRNA PCR products (~657 bp) for five K. pneumoniae isolates (one from each treatment stage), together with positive (K. pneumoniae ATCC 13883) and negative (sterile water) controls.
2. **A separate gel image** showing a subset of PCR products with a 100 bp DNA ladder for size verification.

These figures have been added as Supporting Figure S1, with an accompanying figure legend describing the experimental conditions and interpretation. The figure is cited in the manuscript text at the appropriate location (Molecular Characterization subsection) and is included in the Supporting Information file.

**[Location:** Supporting Information; cited in Results and Discussion, Molecular Characterization subsection]

**Summary of Revisions**

| # | Reviewer Concern | Action Taken |
| --- | --- | --- |
| 1 | Terminology: viable vs. culturable distinction | Revised Terminology Note to explicitly address VBNC caveat; clarified that culturability is a proxy rather than synonym for viability |
| 2 | Table abbreviations undefined | Added comprehensive footnotes to all tables (Tables 1-7) defining abbreviations (n, CFU, PA, %, etc.) |
| 3 | Reference formatting verification | Performed final check of all 40 references for compliance with PLOS ONE Vancouver style; verified author lists, journal abbreviations, DOIs, consistency |
| 4 | Supporting molecular figures | Added Supporting Figure S1 with agarose gel images of 16S rRNA PCR products; included figure legend |

**Additional Editorial Revisions**

In addition to the specific reviewer-requested changes, we have made the following minor corrections:

1. **Scientific precision:** Throughout the manuscript, we have refined language to ensure that claims are appropriately qualified and that the exploratory nature of the study is consistently communicated.
2. **Formatting consistency:** We have verified that all scientific nomenclature is correctly italicized (e.g., *Escherichia coli*, *Klebsiella pneumoniae*), that unit abbreviations are standardized (e.g., mL, CFU, mg/L), and that decimal alignments are consistent.
3. **Figure citation:** Supporting Figure S1 has been cited appropriately in the text where molecular results are discussed.
4. **Supporting Information:** The Supporting Information file has been updated to include the new Supporting Figure S1 and to verify that all supporting tables are correctly referenced in the main text.

**Conclusion**

We are grateful for the thorough and constructive review provided by Reviewer #3. All remaining comments have been addressed in this revision, and we believe the manuscript is now ready for final acceptance in PLOS ONE. We hope that the reviewers and editors find our responses satisfactory and that the manuscript meets the journal's high standards for publication.

Sincerely,

Dr. Hazem Aqel (Corresponding Author)
Basic Medical Sciences Department
College of Medicine, Al-Balqa Applied University
Salt 19117, Jordan
Email: [hazem.aqel@bau.edu.jo](mailto:hazem.aqel@bau.edu.jo)

On behalf of all co-authors
