# Supplementary material for: Persistence of viable opportunistic pathogens in a multi-stage natural wastewater treatment system
Source: PLoS One. 2026 Jul 27;21(7):e0354338. doi: 10.1371/journal.pone.0354338 (PMC13405070; doi:10.1371/journal.pone.0354338)
Supplement: Peer review history

# Peer review: Decision Letter 0

**DOI:** 10.1371/journal.pone.0354338.r001

**Authors:** Muhammad Shahid

7 Apr 2026

PONE-D-26-01733

Persistence of Viable Opportunistic Pathogens in a Multi-Stage Natural Wastewater Treatment System

PLOS One

Dear Dr. Aqel,

Thank you for submitting your manuscript to PLOS ONE. After careful consideration, we feel that it has merit but does not fully meet PLOS ONE’s publication criteria as it currently stands. Therefore, we invite you to submit a revised version of the manuscript that addresses the points raised during the review process.

Please submit your revised manuscript by May 22 2026 11:59PM. If you will need more time than this to complete your revisions, please reply to this message or contact the journal office at plosone@plos.org. When you're ready to submit your revision, log on to https://www.editorialmanager.com/pone/ and select the 'Submissions Needing Revision' folder to locate your manuscript file.

Please include the following items when submitting your revised manuscript:

- A letter that responds to each point raised by the academic editor and reviewer(s). You should upload this letter as a separate file labeled 'Response to Reviewers'.
- A marked-up copy of your manuscript that highlights changes made to the original version. You should upload this as a separate file labeled 'Revised Manuscript with Track Changes'.
- An unmarked version of your revised paper without tracked changes. You should upload this as a separate file labeled 'Manuscript'.

If you would like to make changes to your financial disclosure, please include your updated statement in your cover letter. Guidelines for resubmitting your figure files are available below the reviewer comments at the end of this letter.

If applicable, we recommend that you deposit your laboratory protocols in protocols.io to enhance the reproducibility of your results. Protocols.io assigns your protocol its own identifier (DOI) so that it can be cited independently in the future. For instructions see: https://journals.plos.org/plosone/s/submission-guidelines#loc-laboratory-protocols. Additionally, PLOS ONE offers an option for publishing peer-reviewed Lab Protocol articles, which describe protocols hosted on protocols.io. Read more information on sharing protocols at https://plos.org/protocols?utm_medium=editorial-email&utm_source=authorletters&utm_campaign=protocols.

As the corresponding author, your ORCID iD is verified in the submission system and will appear in the published article. PLOS supports the use of ORCID, and we encourage all coauthors to register for an ORCID iD and use it as well. Please encourage your coauthors to verify their ORCID iD within the submission system before final acceptance, as unverified ORCID iDs will not appear in the published article. *Only*  the individual author can complete the verification step; PLOS staff *cannot*  verify ORCID iDs on behalf of authors.

We look forward to receiving your revised manuscript.

Kind regards,

Muhammad Shahid, PhD

Academic Editor

PLOS One

Journal Requirements:

When submitting your revision, we need you to address these additional requirements.

1. Please ensure that your manuscript meets PLOS ONE's style requirements, including those for file naming. The PLOS ONE style templates can be found at

https://journals.plos.org/plosone/s/file?id=wjVg/PLOSOne_formatting_sample_main_body.pdf and

https://journals.plos.org/plosone/s/file?id=ba62/PLOSOne_formatting_sample_title_authors_affiliations.pdf

2. In your Methods section, please provide additional information regarding the permits you obtained for the work. Please ensure you have included the full name of the authority that approved the field site access and, if no permits were required, a brief statement explaining why.

3. Thank you for stating the following in the Acknowledgments Section of your manuscript:

“The author(s) received no specific funding for this work.”

We note that you have provided funding information that is not currently declared in your Funding Statement. However, funding information should not appear in the Acknowledgments section or other areas of your manuscript. We will only publish funding information present in the Funding Statement section of the online submission form.

Please remove any funding-related text from the manuscript and let us know how you would like to update your Funding Statement. Currently, your Funding Statement reads as follows:

“The author(s) received no specific funding for this work.”

Please include your amended statements within your cover letter; we will change the online submission form on your behalf.

4. We note that Figures 1 and 2 in your submission contain satellite images which may be copyrighted. All PLOS content is published under the Creative Commons Attribution License (CC BY 4.0), which means that the manuscript, images, and Supporting Information files will be freely available online, and any third party is permitted to access, download, copy, distribute, and use these materials in any way, even commercially, with proper attribution. For these reasons, we cannot publish previously copyrighted maps or satellite images created using proprietary data, such as Google software (Google Maps, Street View, and Earth). For more information, see our copyright guidelines: http://journals.plos.org/plosone/s/licenses-and-copyright.

We require you to either (1) present written permission from the copyright holder to publish these figures specifically under the CC BY 4.0 license, or (2) remove the figures from your submission:

1. You may seek permission from the original copyright holder of Figure(s) [#] to publish the content specifically under the CC BY 4.0 license.

We recommend that you contact the original copyright holder with the Content Permission Form (http://journals.plos.org/plosone/s/file?id=7c09/content-permission-form.pdf) and the following text:

“I request permission for the open-access journal PLOS ONE to publish XXX under the Creative Commons Attribution License (CCAL) CC BY 4.0 (http://creativecommons.org/licenses/by/4.0/). Please be aware that this license allows unrestricted use and distribution, even commercially, by third parties. Please reply and provide explicit written permission to publish XXX under a CC BY license and complete the attached form.”

Please upload the completed Content Permission Form or other proof of granted permissions as an "Other" file with your submission.

In the figure caption of the copyrighted figure, please include the following text: “Reprinted from [ref] under a CC BY license, with permission from [name of publisher], original copyright [original copyright year].”

2. If you are unable to obtain permission from the original copyright holder to publish these figures under the CC BY 4.0 license or if the copyright holder’s requirements are incompatible with the CC BY 4.0 license, please either i) remove the figure or ii) supply a replacement figure that complies with the CC BY 4.0 license. Please check copyright information on all replacement figures and update the figure caption with source information. If applicable, please specify in the figure caption text when a figure is similar but not identical to the original image and is therefore for illustrative purposes only.

The following resources for replacing copyrighted map figures may be helpful:

USGS National Map Viewer (public domain): http://viewer.nationalmap.gov/viewer/

The Gateway to Astronaut Photography of Earth (public domain): http://eol.jsc.nasa.gov/sseop/clickmap/

Maps at the CIA (public domain): https://www.cia.gov/library/publications/the-world-factbook/index.html and https://www.cia.gov/library/publications/cia-maps-publications/index.html

NASA Earth Observatory (public domain): http://earthobservatory.nasa.gov/

Landsat: http://landsat.visibleearth.nasa.gov/

USGS EROS (Earth Resources Observatory and Science (EROS) Center) (public domain): http://eros.usgs.gov/#

Natural Earth (public domain): http://www.naturalearthdata.com/

5. We notice that your supplementary tables are included in the manuscript file. Please remove them and upload them with the file type 'Supporting Information'. Please ensure that each Supporting Information file has a legend listed in the manuscript after the references list.

6. Please include captions for your Supporting Information files at the end of your manuscript, and update any in-text citations to match accordingly. Please see our Supporting Information guidelines for more information: http://journals.plos.org/plosone/s/supporting-information.

7. If the reviewer comments include a recommendation to cite specific previously published works, please review and evaluate these publications to determine whether they are relevant and should be cited. There is no requirement to cite these works unless the editor has indicated otherwise.

Additional Editor Comments:

Dear Dr. Aqel,

The manuscirpt titled 'Persistence of Viable Opportunistic Pathogens in a Multi-Stage Natural Wastewater Treatment System' is an interesting research study; however, reveiwers have reaised serious concerns about the presentation and analysis of data along with methodological aspects of the study. I may reconsider my decition should be authors incoporate substainail improvements suggested by reviewers.

[Note: HTML markup is below. Please do not edit.]

Reviewers' comments:

Reviewer's Responses to Questions

**Comments to the Author**

1. Is the manuscript technically sound, and do the data support the conclusions?

The manuscript must describe a technically sound piece of scientific research with data that supports the conclusions. Experiments must have been conducted rigorously, with appropriate controls, replication, and sample sizes. The conclusions must be drawn appropriately based on the data presented.

Reviewer #1: Partly

Reviewer #2: Yes

Reviewer #3: Partly

**********

2. Has the statistical analysis been performed appropriately and rigorously?

Reviewer #1: I Don't Know

Reviewer #2: No

Reviewer #3: N/A

**********

3. Have the authors made all data underlying the findings in their manuscript fully available?

The PLOS Data policy requires authors to make all data underlying the findings described in their manuscript fully available without restriction, with rare exception (please refer to the Data Availability Statement in the manuscript PDF file). The data should be provided as part of the manuscript or its supporting information, or deposited to a public repository. For example, in addition to summary statistics, the data points behind means, medians and variance measures should be available. If there are restrictions on publicly sharing data—e.g. participant privacy or use of data from a third party—those must be specified.

Reviewer #1: No

Reviewer #2: Yes

Reviewer #3: No

**********

4. Is the manuscript presented in an intelligible fashion and written in standard English?

PLOS ONE does not copyedit accepted manuscripts, so the language in submitted articles must be clear, correct, and unambiguous. Any typographical or grammatical errors should be corrected at revision, so please note any specific errors here.

Reviewer #1: No

Reviewer #2: Yes

Reviewer #3: No

**********

5. Review Comments to the Author

Please use the space provided to explain your answers to the questions above. You may also include additional comments for the author, including concerns about dual publication, research ethics, or publication ethics. (Please upload your review as an attachment if it exceeds 20,000 characters)

Reviewer #1: Manuscript title: Persistence of Viable Opportunistic Pathogens in a Multi-Stage Natural WastewaterTreatment System

Manuscript Number: PONE-D-26-01733

This research aimed to determine the bacterial population across different stages of a wastewater treatment system using culture-dependent techniques. The study represents original work on the microbiological and physicochemical characterization of the wastewater treatment plant in Wadi Hanifa, Riyadh, Saudi Arabia. Overall, the manuscript meets the journal’s requirements. It can be considered for publication in PLOS One after major and comperhensive revisions and careful attention to all reviewer comments. The following major and specific comments are provided to help improve the quality and clarity of the manuscript.

Major comments

- The authors relied on the API system as a classical biochemical method for the identification of bacterial isolates. However, more recent automated biochemical identification systems, such as Biolog and VITEK, are now widely used. Therefore, the authors should clearly justify and highlight the use of the API system in the Introduction section, explaining the rationale for selecting this method over more advanced automated techniques.

-The authors focused on determining the total culturable bacterial population in the collected wastewater. However, it is unclear why fecal indicator bacteria (FIB), particularly Escherichia coli, were not detected in the downstream samples, while other microorganisms were still present. It is well established that E. coli typically represents 90–95% of FIB in wastewater. The authors are encouraged to clarify this observation and provide a possible explanation. Additionally, the use of selective media specifically targeting E. coli might have yielded different results, and this methodological aspect should be discussed.

- The number of isolates recovered in this study is too limited to support reliable conclusions regarding the distribution, fate, and persistence of pathogenic bacteria across the different treatment stages. Drawing interpretations from such a small dataset may compromise the validity of the findings and weakens the statistical analysis. Moreover, the authors must clearly report in the Materials and Methods section the total number of isolates obtained, as well as the number recovered from each individual treatment stage. This information is essential for transparency and is currently unclear in the manuscriptز

- The Results and Discussion section lacks depth and clarity. It requires significant revision to present the findings more clearly, strengthen the interpretation of the results, and improve the overall scientific quality of the manuscriptز

Specific comments

-Line 23, write the word “ viable” in nonitalic due to this not microorganisms name and revise it in the whole manuscript.

- Line 55, transfer this sentence into materials and methods section.

- Line 85, merge two parts about Study area and sampling design in one part, also, delect Fig 1. where figure 2 is enough.

- Line 90, The statement “Although sampling was conducted in 2015” raises some concern, as this means the samples were collected approximately 11 years ago. The authors should clarify whether this date is correct. If accurate, they should justify the relevance of using samples collected such a long time ago, considering possible changes in wastewater characteristics and treatment processes over time. Also, it will be useful if the authors mentined number of population that plant serve them.

-Line 100, Please rewrite the description of the sampling locations in the form of clear, complete sentences rather than listing them. Additionally, it is important to specify whether a final disinfection step is applied in the treatment process. The authors should also clarify how the treated wastewater (final effluent) is used after discharge.

-Line 118, Please provide appropriate references for the sampling collection procedure and transportation methods to ensure that they follow standard protocols.

- Line 124, Chemical Oxygen Demand (COD) is considered an important parameter for indicating the organic load in wastewater. It would be useful to include COD determination in the analysis. If it was measured, the authors should report the results; if not, they should justify its omission.

-Line 151, the name of antibiotic should be mentioned in this section.

- Based on Sequence analsyis for Klebsiella pneumoniae, the accession number should be added in materials and methods or results section.

-line 179, The authors neglected to discuss the remaining physicochemical parameters, such as pH, temperature, and electrical conductivity (EC). These parameters are important for understanding wastewater characteristics and their potential influence on microbial communities. A more detailed discussion of these results is recommended.

-Line 198, The authors should clarify the regulatory limits for wastewater reuse in irrigation purposes. Bacterial count (BA count) alone is generally not considered an appropriate indicator for reuse standards, as most national and international guidelines are based primarily on fecal coliforms and/or Escherichia coli counts. Therefore, the authors are encouraged to discuss the applicable standards and justify the parameters used to assess the suitability of the treated wastewater for irrigation.

- Line 219,could be clarify how Confidence % calculaed for each isolate and this should be clear in Materials and Methods section.

Reviewer #2: Greetings

Good work. But kindly edit the following points:

Major Comments

Methodological clarification:

The sampling design and experimental workflow need to be described more clearly. The authors should provide additional details about the sampling frequency, the number of replicates, and the distribution of sampling points across the different stages of the treatment system.

Lack of molecular confirmation:

The identification of bacterial isolates appears to rely mainly on culture-based methods. Including molecular confirmation, such as PCR or sequencing of the 16S rRNA gene or species-specific markers, would increase the reliability of species identification, particularly for clinically important pathogens like Klebsiella pneumoniae.

Antimicrobial resistance analysis:

Although antimicrobial susceptibility testing is mentioned, the resistance patterns are not discussed in sufficient detail. A clearer presentation and interpretation of these resistance profiles, along with their potential public health implications, would strengthen the manuscript.

Data presentation and statistical analysis:

Some of the results would benefit from clearer presentation and more detailed statistical analysis. The manuscript should specify the statistical tests used, as well as the confidence levels and the rationale for the correlations reported.

Depth of discussion:

The discussion section could be strengthened by comparing the findings more thoroughly with recent studies on pathogen persistence in natural wastewater treatment systems and nature-based treatment approaches. Expanding on the implications for wastewater reuse and environmental risk assessment would also improve the manuscript.

Minor Comments

The language should be revised in several sections to improve clarity and readability.

Bacterial names should be formatted consistently in italics throughout the manuscript.

Abbreviations should be written in full when they first appear in the text.

Some figures and tables would benefit from clearer legends and units.

Minor grammatical corrections are needed across the manuscript.

Kind regards.

Reviewer #3: Overall Assessment

This manuscript examines the persistence of viable opportunistic pathogens in a multi-stage natural wastewater treatment system in an arid region. The culture-based approach is a real strength, especially since much of the existing research relies on molecular techniques that don’t confirm whether bacteria are alive. The finding that Klebsiella pneumoniae persists even when Escherichia coli is removed is significant, as it highlights the potential limitations of relying solely on fecal indicators for monitoring. The paper is generally well-written and well-organized, but some key aspects—like sample size, the time covered, statistical analysis, and how the results are interpreted—need closer attention.

Strengths

Tackles important public health questions for water-scarce regions.

Culture-based methods confirm the presence of live bacteria, going beyond what molecular studies can show.

Multi-stage sampling gives a good picture of how microbial populations change through the treatment process.

The comparison between traditional fecal indicators and opportunistic pathogens highlights gaps in existing monitoring methods.

Good integration of both physicochemical and microbiological data.

Ethical approval and quality control procedures are clearly described.

Major Issues

Title Page

The label “Manuscript Title:” should be removed. PLOS ONE guidelines say the title should appear without section headings.

There’s an extra space in the author list after the corresponding author symbol; this should be fixed.

ORCID identifiers need to be in URL format, not just “ORCID ID."

Only the email address for the corresponding author should be included, not the phone number.

Abstract

The abstract is clear and organized, but the study objective should be stated more directly.

The use of culture-based identification should be described carefully to avoid giving a false sense of taxonomic certainty.

Given the small sample size, the findings should be described as exploratory.

When reporting percentages (like 44.4% or 62.5%), it would help to include the actual numbers.

The correlation (Kendall’s τ = 0.84) should be interpreted with caution due to the small sample size.

The statement that multidrug-resistant Klebsiella pneumoniae is not eliminated should be softened (e.g., “under the conditions examined”).

The limitations of indicator-based monitoring should be described as specific to this system, not generalized.

The keywords are suitable, but adding “antimicrobial resistance” would help with indexing.

Introduction

The background is clear and narrows appropriately to the study’s context.

The research gap (viable opportunistic pathogen persistence in nature-based treatment) should be stated more explicitly and earlier.

The limitations of fecal indicator bacteria are discussed, but the implications for risk assessment in wastewater reuse could be clearer.

The focus on Klebsiella pneumoniae is reasonable, but the rationale could be strengthened by discussing its environmental persistence and its relevance to irrigation exposure.

Citing more regional or local studies, especially from Saudi Arabia or similar arid environments, would improve context.

The discussion of DNA-based detection limitations justifies the use of culture-based methods.

The objectives are clear and aligned with the research gap, but adding a sentence about the public health relevance of viability-based monitoring would increase the impact.

The introduction should end with a clear statement of the study’s expectations or hypotheses.

Methods

The study area and sampling design are clearly described, but it would help to explain why samples were collected in November 2015 and to address possible seasonal variation.

The total number of isolates (n = 18) is quite small. The criteria for selecting isolates should be clarified.

It should be clear whether sampling was done on one or multiple days and whether triplicates were technical or biological replicates.

While the microbiological methods are appropriate, using only blood agar and MacConkey agar could introduce bias, and this should be acknowledged.

The manuscript should specify how many isolates were characterized and how many were confirmed via 16S rRNA sequencing.

The antimicrobial susceptibility testing follows CLSI guidelines, but the list of antibiotics tested should be included for reproducibility.

Statistical analysis is generally appropriate, but justification is needed for using linear regression and Cochran–Armitage tests with such a small sample size.

Quality control is described well, but more detail on calibration and contamination control during sampling and transport would strengthen this section.

Results and Discussion

The section is well organized and links physicochemical changes, bacterial reduction, and antimicrobial resistance findings logically.

Some subsections could be merged for clarity and to avoid overlap (for example, combining the correlation and diversity of subsections).

The 1.1 log₁₀ reduction in bacterial counts is clearly stated, but comparisons to WHO targets should specify that these refer to pathogen removal, not total culturable bacteria.

There is some redundancy between tables and text; the text should focus on the main findings.

The persistence of Klebsiella pneumoniae is an important result, but conclusions about indicator-based monitoring should be system-specific.

Percentages should always be accompanied by absolute numbers.

Correlation results should be interpreted cautiously due to the limited number of samples.

Statements about wastewater systems as global reservoirs of antimicrobial resistance should be moderated, given the limited data.

The conclusion that physical removal is the main driver of bacterial reduction is reasonable, but it should mention other possible mechanisms.

The public health implications are somewhat overstated, especially given that no quantitative microbial risk assessment (QMRA) was done.

The discussion should more clearly acknowledge the small sample size, the single time point, and the limitations of culture-based methods.

Comparison with previous studies is present but could be expanded, especially with studies from similar climates or treatment systems.

Conclusion

The conclusion is clear but somewhat overstated.

The statement that natural treatment systems “do not eliminate” pathogens should be softened to reflect the study’s limitations.

Suggestions for adding monitoring or treatment steps are helpful but should note practical limitations and the limited evidence.

The conclusion should state clearly that the findings are preliminary, and that larger, long-term studies are needed.

Data Availability

At present, the manuscript does not fully meet PLOS’s data availability requirements. Key underlying data—such as raw microbiological counts, isolate-level information, and detailed results from antimicrobial susceptibility testing—are either missing or not provided in enough detail within the manuscript or as supplementary materials. For the sake of transparency and reproducibility, these data should be made fully accessible, either by including them as supplementary files or by sharing them through a public repository.

Minor Issues

There are minor grammatical and typographical errors throughout; careful proofreading is recommended.

Terminology (e.g., “viable bacteria” vs. “culturable bacteria”) should be used consistently.

Tables and figures would benefit from better formatting and clearer captions.

Some references are duplicated or repeated—these should be checked carefully.

Abbreviations should be defined at first use and used consistently.

Statistical reporting (e.g., p-values, decimal places) should be consistent throughout.

Reducing repetition between the Results and Discussion sections would strengthen the manuscript.

Supporting tables are clear and useful; key trends could be highlighted in the main text for easier interpretation. The clustering table is informative, but its relevance to pathogen persistence could be explained briefly.

All supporting files (e.g., supplementary tables, raw data, detailed methods, and additional figures) should be properly prepared, clearly labeled, and submitted alongside the manuscript to ensure transparency, reproducibility, and compliance with journal requirements.

Author's contributions are clear; you might add that all authors approved the final version.

References

Most references are relevant and well-formatted, but please check for duplicates (for example, 16/34, 17/35, 29/37, 33/36) and make sure all are cited correctly.

Recommendation: Major Revision

**********

6. PLOS authors have the option to publish the peer review history of their article (what does this mean?). If published, this will include your full peer review and any attached files.

If you choose “no”, your identity will remain anonymous but your review may still be made public.

**Do you want your identity to be public for this peer review?**  For information about this choice, including consent withdrawal, please see our Privacy Policy.

Reviewer #1: **Yes:** Mohamed Azab El-Liethy

Reviewer #2: No

Reviewer #3: No

**********

[NOTE: If reviewer comments were submitted as an attachment file, they will be attached to this email and accessible via the submission site. Please log into your account, locate the manuscript record, and check for the action link "View Attachments". If this link does not appear, there are no attachment files.]

To ensure your figures meet our technical requirements, please review our figure guidelines: https://journals.plos.org/plosone/s/figures

You may also use PLOS’s free figure tool, NAAS, to help you prepare publication quality figures: https://journals.plos.org/plosone/s/figures#loc-tools-for-figure-preparation.

NAAS will assess whether your figures meet our technical requirements by comparing each figure against our figure specifications.

# Author comment: Author response to Decision Letter 1

**DOI:** 10.1371/journal.pone.0354338.r002

19 Apr 2026

Dear Dr. Shahid,

We thank the Academic Editor and all three reviewers for their thorough and constructive evaluation of our manuscript. The comments have substantially improved the quality, clarity, and scientific rigor of the work. Below, we provide a point-by-point response to each comment. All changes are highlighted in the Revised Manuscript with Track Changes file. Reviewer comments appear in italics; our responses are in plain text.

Journal Requirements and Editor Comments

Journal Requirement 1: PLOS ONE style compliance

Please ensure that your manuscript meets PLOS ONE's style requirements.

Response: The manuscript has been reformatted to comply with PLOS ONE style requirements. The title page has been revised to remove the 'Manuscript Title:' label, ORCID identifiers are now provided in URL format, and the phone number has been removed from the author block, retaining only the corresponding author's email address. An extra space after the corresponding author symbol has also been corrected.

Journal Requirement 2: Permits for field site access

Please provide additional information regarding the permits you obtained for the work.

Response: We have added the following statement to the Methods section (Study area and sampling design): 'Access to the Wadi Hanifa treatment corridor for environmental water sampling was granted under the research agreement with King Abdullah International Medical Research Centre (KAIMRC; reference number KAIMRC/RFC/0092/17); no additional field permits were required as samples were collected from the surface of a managed public treatment waterway.'

Journal Requirement 3: Funding statement

Funding information should not appear in the Acknowledgments section. Please confirm the Funding Statement.

Response: We confirm that no specific funding was received for this work. The Funding Statement reads: 'The author(s) received no specific funding for this work.' No funding information appears in the Acknowledgments or elsewhere in the manuscript body.

Journal Requirement 4: Copyright of satellite images (Figures 1 and 2)

Figures 1 and 2 contain satellite images from Google Earth which may be copyrighted and cannot be published under CC BY 4.0 without permission.

Response: We acknowledge this important concern. Figures 1 and 2 (satellite imagery from Google Earth Pro/Maxar Technologies) have been removed from the revised manuscript. We note that Figure 2 (the schematic layout of sampling locations) was the primary informational figure, and we have retained the descriptive text of the sampling design within the Methods section. We are currently exploring the use of open-source map resources (e.g., OpenStreetMap, NASA Earth Observatory) to prepare a compliant replacement figure and will include it upon final acceptance if permission or a compliant alternative is secured.

Journal Requirement 5 & 6: Supporting Information files

Supporting tables should be removed from the manuscript file and uploaded as separate Supporting Information files with captions listed in the manuscript after the references.

Response: Tables S1 and S2 have been removed from the main manuscript text and are provided as separate Supporting Information files. Captions for both supporting tables are now listed in the manuscript after the References section under a 'Supporting Information' heading, as required.

Journal Requirement 7: Duplicate references

Please review and evaluate publications recommended by reviewers and check for duplicate references.

Response: We have reviewed the reference list and identified duplicate entries (references 16/34, 17/35, 29/37, 33/36 in the original). The duplicate entries have been removed from the revised manuscript. The reference list has been renumbered accordingly, and all in-text citations have been updated.

Editor Comments

The manuscript has merit but does not fully meet PLOS ONE's publication criteria. Reviewers have raised serious concerns about the presentation and analysis of data along with methodological aspects.

Response: We thank the Academic Editor for this constructive assessment. We have made substantial revisions to address all concerns raised by the three reviewers, including: expanding the Methods section with greater detail on isolate selection criteria, statistical justification, and antibiotic panel; moderating overstated conclusions to reflect the exploratory and preliminary nature of the findings; adding explicit acknowledgement of study limitations (single time point, small sample size, culture-based bias); improving the discussion with expanded comparison to the literature; and correcting all minor language and formatting issues. We describe each change in detail below.

Reviewer #1 (Mohamed Azab El-Liethy) – Major Comments

R1-Major 1: Justification of API system

The authors relied on the API system. More recent automated systems such as Biolog and VITEK are now widely used. The authors should justify the use of the API system in the Introduction.

Response: We thank the reviewer for this valid point. We have added the following justification in the Introduction: 'The API system (bioMérieux) was selected for biochemical identification because it provides a standardized, cost-effective, and well-validated approach for environmental isolate identification, with an established database widely used in both clinical and environmental microbiology. This choice was also consistent with the resources available at the time of sample processing (2015).' We acknowledge that automated platforms such as VITEK 2 or MALDI-TOF MS would provide faster and potentially more accurate identification and should be considered in future studies.

R1-Major 2: Absence of E. coli in downstream samples

It is unclear why E. coli was not detected in downstream samples while other organisms were still present. The use of selective media specifically targeting E. coli might have yielded different results.

Response: This is an important point. We have expanded the discussion to address this observation explicitly. In the revised manuscript, we identify three plausible explanations: (i) the use of non-selective BA and MAC agar rather than chromogenic media (e.g., CHROMagar Orientation or Brilliance E. coli/Coliform agar) may have resulted in underestimation of E. coli in downstream samples; (ii) E. coli is known to be more susceptible to environmental stressors (elevated DO, reduced organic matter, UV) than non-enteric facultative Gram-negatives; and (iii) the very low abundance at downstream stages may have fallen below the detection limit of our plating approach. We have added this discussion and acknowledged the use of non-selective media as a methodological limitation.

R1-Major 3: Limited number of isolates

The number of isolates is too limited to support reliable conclusions. The total number of isolates and the number from each stage must be clearly reported in Materials and Methods.

Response: We agree that this is a significant limitation. We have (i) clearly stated in the Methods (Bacterial isolation and enumeration) that a total of 18 morphologically distinct colonies were selected across all five treatment stages (approximately 3–4 isolates per stage), and have described the morphotype-based selection criteria; (ii) explicitly framed the study as exploratory and preliminary throughout the manuscript, including in the Abstract, Results and Discussion, and Conclusions; and (iii) added a statement in the Conclusions recommending larger, multi-season studies with expanded isolate collections to validate these findings.

R1-Major 4: Results and Discussion depth

The Results and Discussion section lacks depth and clarity and requires significant revision.

Response: The Results and Discussion section has been substantially revised. Key improvements include: (i) expanded interpretation of physicochemical data (pH, temperature, EC) and their relevance to microbial communities; (ii) more detailed mechanistic discussion of K. pneumoniae persistence; (iii) expanded comparison with recent studies from similar climates and systems; (iv) cautious language throughout to reflect the small sample size; and (v) reduced redundancy between tables and text.

R1-Specific 1: Italics for 'viable'

Line 23: write the word 'viable' in non-italic throughout the manuscript.

Response: Corrected throughout the manuscript. The word 'viable' is not a microorganism name and has been changed to roman text.

R1-Specific 2: Sentence on Wadi Hanifa statistics

Line 55: transfer this sentence to the Materials and Methods section.

Response: The sentence describing the volume received by the Wadi Hanifa system (300,000 m³ day⁻¹) has been moved to the Study area and sampling design subsection of Methods. We have also added that the system serves approximately 7 million people in the greater Riyadh metropolitan area.

R1-Specific 3: Merge study area subsections and remove Figure 1

Line 85: merge the two parts about Study area and sampling design into one and delete Fig 1 as Figure 2 is sufficient.

Response: The two subsections (Study site description and Sampling locations) have been merged into a single section titled 'Study area and sampling design.' Figure 1 & Figure 2 has been removed (also addressed under Journal Requirement 4 regarding copyright).

R1-Specific 4: Sampling date and population served

Line 90: The 2015 sampling date needs justification for current relevance. Also, the number of people served by the plant should be mentioned.

Response: We have added an explanation that the November 2015 sampling date represents stable dry-season operating conditions and that the treatment configuration has remained consistent. We have also added a note on the population served (~7 million, greater Riyadh area). We acknowledge that 10-year-old data are a limitation and have framed the study accordingly.

R1-Specific 5: Sampling location description and effluent use

Line 100: Rewrite sampling location descriptions as complete sentences. Clarify whether a final disinfection step is applied and how the final effluent is used.

Response: The bullet-point list of sampling locations has been replaced with full sentences in the revised manuscript. We have added that no terminal chlorination step is applied and that the final effluent is used for landscape irrigation and green belt maintenance in Riyadh.

R1-Specific 6: References for sample collection procedure

Line 118: Provide appropriate references for sample collection and transportation methods.

Response: A reference to the Standard Methods for the Examination of Water and Wastewater (APHA, 2023; reference 32) has been added to the sample collection paragraph.

R1-Specific 7: COD determination

Line 124: COD is an important parameter. If not measured, justify its omission.

Response: COD was not measured in this study. We have added the following statement in the Physicochemical analysis section: 'Chemical oxygen demand (COD) was not measured in this study; determination of COD would have required a separate sampling and analytical setup that was not available at the time of field collection. Future studies should include COD as a standard organic loading parameter.'

R1-Specific 8: Antibiotic names in Methods

Line 151: the names of antibiotics tested should be mentioned in this section.

Response: The full list of eight antimicrobial agents tested (with drug class and disk concentration) has been added to the Antimicrobial susceptibility testing subsection: ampicillin (10 µg), amoxicillin-clavulanate (20/10 µg), ceftazidime (30 µg), gentamicin (10 µg), ciprofloxacin (5 µg), trimethoprim-sulfamethoxazole (1.25/23.75 µg), amikacin (30 µg), and meropenem (10 µg).

R1-Specific 9: Discussion of pH, temperature, and EC

Line 179: The authors neglected to discuss pH, temperature, and EC.

Response: A dedicated discussion of all physicochemical parameters has been added. We now address pH (modest increase from 7.4 to 7.8, consistent with photosynthetic activity in bio-cells), temperature (stable at 24.8–25.5°C, reflecting dry-season conditions), and EC (minimal variation, indicating limited alteration of dissolved salts). We discuss the implications of the stability of these parameters for the observed microbial community changes.

R1-Specific 10: Regulatory limits for wastewater reuse

Line 198: The authors should clarify the regulatory limits for wastewater reuse and justify the parameters used to assess suitability for irrigation.

Response: We have revised this paragraph to clarify that the WHO ≥5-log reduction target refers to pathogen-specific reductions (e.g., for helminth eggs, E. coli, and rotavirus) rather than total culturable bacterial counts, and that the comparison is provided for context only. We note that most national and international guidelines are based primarily on E. coli/fecal coliforms and discuss this in the context of the limitations of indicator-based monitoring highlighted by our findings.

R1-Specific 11: API confidence calculation

Line 219: How is API confidence % calculated? This should be clarified in Methods.

Response: We have added an explanation in the Phenotypic, biochemical, and molecular characterization subsection: 'Identification was accepted at ≥80% confidence using the apiweb™ database; the percentage confidence reflects the probability of the given identification based on the biochemical reaction profile.' We have also added a footnote to Table 3 to this effect.

Reviewer #2 – Major Comments

R2-Major 1: Sampling design clarity

The sampling design and experimental workflow need more detail: sampling frequency, number of replicates, distribution of sampling points.

Response: We have clarified in the Methods that: (i) sampling was conducted on a single occasion (November 2015), representing a single time point; (ii) triplicate technical replicates were collected at each of the five sampling locations; (iii) a total of 18 morphologically distinct isolates were selected across all stages. We acknowledge the single time-point as a study limitation and recommend repeated sampling in future studies.

R2-Major 2: Molecular confirmation

Identification relies mainly on culture-based methods. Including molecular confirmation such as 16S rRNA sequencing would increase reliability.

Response: We have clarified in the Methods that representative K. pneumoniae isolates from each treatment stage (n = 5) were confirmed by partial 16S rRNA gene sequencing (∼1,400 bp) with ≥99% identity to reference sequences, as was described in the original manuscript.

R2-Major 3: Antimicrobial resistance analysis

Resistance patterns are not discussed in sufficient detail. Clearer presentation and interpretation with public health implications would strengthen the manuscript.

Response: The antimicrobial resistance section has been expanded. We now discuss: (i) the significance of universal ampicillin resistance as intrinsic to K. pneumoniae; (ii) the elevated resistance to ceftazidime and amoxicillin-clavulanate as suggestive of possible ESBL activity; (iii) the retention of susceptibility to amikacin and meropenem as reassuring for clinical management; and (iv) the public health implications of viable MDR K. pneumoniae in irrigation effluent, while noting that quantitative risk assessment was not performed.

R2-Major 4: Data presentation and statistics

Results would benefit from clearer presentation and more detailed statistical analysis. Specify statistical tests, confidence levels, and rationale for correlations.

Response: All statistical tests are now explicitly stated in the Methods and referenced in the Results. Justification for the choice of Kendall’s τ, linear regression, and Cochran–Armitage tests given the small sample size has been added. Confidence levels and p-values are reported consistently throughout.

R2-Major 5: Depth of discussion

The discussion could be strengthened by comparing findings with recent studies on pathogen persistence in natural wastewater treatment systems.

Response: The discussion has been substantially expanded with comparisons to recent studies from similar arid-region and nature-based treatment contexts, including studies from Saudi Arabia (reference 30) and other arid environments. The implications for QMRA and wastewater reuse policy

Attachment Submitted filename: Response to Reviewers.docx

# Peer review: Decision Letter 1

**DOI:** 10.1371/journal.pone.0354338.r003

**Authors:** Muhammad Shahid

14 May 2026

PONE-D-26-01733R1

Persistence of Viable Opportunistic Pathogens in a Multi-Stage Natural Wastewater Treatment System

PLOS One

Dear Dr. Aqel,

Thank you for submitting your manuscript to PLOS ONE. After careful consideration, we feel that it has merit but does not fully meet PLOS ONE’s publication criteria as it currently stands. Therefore, we invite you to submit a revised version of the manuscript that addresses the points raised during the review process.

The revised version of the manuscript has been significantly improved; however, the reviewers have suggested more changes to be incorporated. Please revise the ms according to the reviewers' comments and submit the revised version

Please submit your revised manuscript by Jun 28 2026 11:59PM. If you will need more time than this to complete your revisions, please reply to this message or contact the journal office at plosone@plos.org. When you're ready to submit your revision, log on to https://www.editorialmanager.com/pone/ and select the 'Submissions Needing Revision' folder to locate your manuscript file.

Please include the following items when submitting your revised manuscript:

- A letter that responds to each point raised by the academic editor and reviewer(s). You should upload this letter as a separate file labeled 'Response to Reviewers'.
- A marked-up copy of your manuscript that highlights changes made to the original version. You should upload this as a separate file labeled 'Revised Manuscript with Track Changes'.
- An unmarked version of your revised paper without tracked changes. You should upload this as a separate file labeled 'Manuscript'.

If you would like to make changes to your financial disclosure, please include your updated statement in your cover letter. Guidelines for resubmitting your figure files are available below the reviewer comments at the end of this letter.

If applicable, we recommend that you deposit your laboratory protocols in protocols.io to enhance the reproducibility of your results. Protocols.io assigns your protocol its own identifier (DOI) so that it can be cited independently in the future. For instructions see: https://journals.plos.org/plosone/s/submission-guidelines#loc-laboratory-protocols. Additionally, PLOS ONE offers an option for publishing peer-reviewed Lab Protocol articles, which describe protocols hosted on protocols.io. Read more information on sharing protocols at https://plos.org/protocols?utm_medium=editorial-email&utm_source=authorletters&utm_campaign=protocols.

As the corresponding author, your ORCID iD is verified in the submission system and will appear in the published article. PLOS supports the use of ORCID, and we encourage all coauthors to register for an ORCID iD and use it as well. Please encourage your coauthors to verify their ORCID iD within the submission system before final acceptance, as unverified ORCID iDs will not appear in the published article. *Only*  the individual author can complete the verification step; PLOS staff *cannot*  verify ORCID iDs on behalf of authors.

We look forward to receiving your revised manuscript.

Kind regards,

Muhammad Shahid, PhD

Academic Editor

PLOS One

Journal Requirements:

If the reviewer comments include a recommendation to cite specific previously published works, please review and evaluate these publications to determine whether they are relevant and should be cited. There is no requirement to cite these works unless the editor has indicated otherwise.

Additional Editor Comments:

The present version of the manuscript has been significantly improved; however, one of the reviewers identified major flaws and therefore I suggest to incorporate the suggested changes.

[Note: HTML markup is below. Please do not edit.]

Reviewers' comments:

Reviewer's Responses to Questions

**Comments to the Author**

1. If the authors have adequately addressed your comments raised in a previous round of review and you feel that this manuscript is now acceptable for publication, you may indicate that here to bypass the “Comments to the Author” section, enter your conflict of interest statement in the “Confidential to Editor” section, and submit your "Accept" recommendation.

Reviewer #1: All comments have been addressed

Reviewer #2: All comments have been addressed

Reviewer #3: (No Response)

**********

2. Is the manuscript technically sound, and do the data support the conclusions?

The manuscript must describe a technically sound piece of scientific research with data that supports the conclusions. Experiments must have been conducted rigorously, with appropriate controls, replication, and sample sizes. The conclusions must be drawn appropriately based on the data presented.

Reviewer #1: Yes

Reviewer #2: Yes

Reviewer #3: Yes

**********

3. Has the statistical analysis been performed appropriately and rigorously?

Reviewer #1: I Don't Know

Reviewer #2: I Don't Know

Reviewer #3: Yes

**********

4. Have the authors made all data underlying the findings in their manuscript fully available?

The PLOS Data policy requires authors to make all data underlying the findings described in their manuscript fully available without restriction, with rare exception (please refer to the Data Availability Statement in the manuscript PDF file). The data should be provided as part of the manuscript or its supporting information, or deposited to a public repository. For example, in addition to summary statistics, the data points behind means, medians and variance measures should be available. If there are restrictions on publicly sharing data—e.g. participant privacy or use of data from a third party—those must be specified.

Reviewer #1: Yes

Reviewer #2: Yes

Reviewer #3: No

**********

5. Is the manuscript presented in an intelligible fashion and written in standard English?

PLOS ONE does not copyedit accepted manuscripts, so the language in submitted articles must be clear, correct, and unambiguous. Any typographical or grammatical errors should be corrected at revision, so please note any specific errors here.

Reviewer #1: Yes

Reviewer #2: Yes

Reviewer #3: Yes

**********

6. Review Comments to the Author

Please use the space provided to explain your answers to the questions above. You may also include additional comments for the author, including concerns about dual publication, research ethics, or publication ethics. (Please upload your review as an attachment if it exceeds 20,000 characters)

Reviewer #1: (No Response)

Reviewer #2: Greetings

The necessary modifications have been appropriately addressed, and the scientific quality of the paper has significantly improved. The revised version is now stronger, clearer, and more suitable for publication.

Kind regards.

Reviewer #3: Review for manuscript entitled: “Persistence of Viable Opportunistic Pathogens in a Multi-Stage Natural Wastewater Treatment System”

Overall Assessment

This revised manuscript is noticeably improved compared with the original version. The authors have addressed many of the earlier concerns by presenting the findings more cautiously, adding important methodological details, and acknowledging the limitations of the study more clearly. The paper now provides a more balanced discussion of viable opportunistic pathogen persistence in a nature-based wastewater treatment system.

A major strength of the study remains in the culture-based approach, which allows assessment of viable (culturable) bacteria rather than relying solely on DNA-based methods that cannot distinguish between live and dead cells. The finding that Klebsiella pneumoniae persisted throughout the treatment system while Escherichia coli was no longer detected downstream is particularly interesting and raises important questions about the limitations of conventional fecal indicators in wastewater reuse monitoring.

Strengths

• The study addresses an important issue related to wastewater reuse and public health in arid and water-scarce regions.

• The culture-based methodology is a valuable strength because it confirms the persistence of viable bacteria.

• The revised introduction explains the rationale and research gap more clearly and now includes a useful hypothesis statement.

• The focus on Klebsiella pneumoniae is better justified in terms of environmental persistence and public health relevance.

• The sampling design and treatment stages are now described more clearly.

• The authors appropriately frame the findings as exploratory and preliminary.

• Additional methodological details, including QA/QC procedures, isolate selection criteria, and antimicrobial susceptibility testing, improve transparency and reproducibility.

• The discussion is more balanced, particularly regarding antimicrobial resistance and the limitations of indicator-based monitoring.

• Study limitations, including the small sample size and culture bias, are acknowledged more openly.

Major Issues

Figure numbering inconsistency

The Methods section still refers to “Fig 1,” although the manuscript indicates that Figure 1 was removed due to copyright concerns. Figure references should be checked carefully and updated consistently throughout the manuscript.

Temporal limitation of the dataset

The additional explanation regarding the use of samples collected in November 2015 is appreciated. However, readers may still question the relevance of older samples in a recently submitted manuscript. Adding a brief statement in the limitations section explaining the continuing value of these data would strengthen the manuscript further.

Interpretation of statistical findings

The revised statistical interpretation is appropriately more cautious than in the original version. However, given the small sample size (five treatment stages and 18 isolates), some statements could still be softened slightly. For example:

“Isolate richness declined by 50% across treatment stages (p = 0.022)”

could be rephrased more conservatively as:

“Observed isolate richness declined by approximately 50% across treatment stages.”

The manuscript should continue emphasizing that the statistical analyses are exploratory and intended to identify trends rather than provide definitive inference.

Public health interpretation

The discussion has improved considerably, but a few statements regarding wastewater systems as reservoirs or dissemination pathways for antimicrobial resistance should still be moderated slightly given the limited isolate number and the absence of molecular resistance characterization.

Supporting Information and raw data availability

Although Supporting Information files are now mentioned, the manuscript still does not clearly indicate whether the raw data underlying the analyses are publicly available. In line with PLOS ONE data availability requirements, the authors should clarify whether the raw microbiological counts, physicochemical measurements, antimicrobial susceptibility data, and sequencing results are included within the Supporting Information files or deposited in a public repository.

The manuscript would also benefit from clearer citation of Supporting Information files within the main text. If sequencing data is available, accession numbers should be included before final submission.

Minor Issues

• A careful proofreading is still recommended to correct a few remaining formatting inconsistencies and typographical errors.

• Terminology should remain consistent throughout the manuscript, particularly regarding “viable,” “culturable,” and “culture-based” bacteria.

• Hyphenation and formatting should be standardized throughout the manuscript (e.g., “multi-stage,” “sand-filtered,” and “culture-based”).

• Statistical reporting and decimal formatting should be checked for consistency.

• Some repetition between the Results and Discussion sections could still be reduced slightly.

• The WHO comparison is now better qualified, but the manuscript should continue clarifying that WHO benchmarks refer to pathogen-specific reductions rather than total culturable bacterial counts.

• The Results and Discussion sections would benefit from additional references to strengthen comparisons with previous studies and better contextualize findings, particularly on pathogen persistence, antimicrobial resistance, and indicator limitations.

• Ensure that GenBank accession numbers are included before final submission.

• The Supporting Information files are now more clearly organized and easier to follow.

• The clustering table is informative, but a brief explanation of its relevance to pathogen persistence within the main text would improve clarity.

• The revised Author Contributions section is appropriate and clearly states that all authors approved the final version of the manuscript.

• The duplicate reference issue appears to have been addressed, although the final reference list should still be checked carefully before submission.

Recommendation

Major Revision

This revised manuscript is considerably stronger than the original submission and addresses many of the previously raised concerns. The remaining issues mainly involve editorial cleanup, consistency, data transparency, and additional moderation of interpretation rather than major methodological flaws. With careful revision and proofreading, the manuscript could become suitable for publication.

**********

7. PLOS authors have the option to publish the peer review history of their article (what does this mean?). If published, this will include your full peer review and any attached files.

If you choose “no”, your identity will remain anonymous but your review may still be made public.

**Do you want your identity to be public for this peer review?**  For information about this choice, including consent withdrawal, please see our Privacy Policy.

Reviewer #1: **Yes:** Mohamed Azab El-Liethy

Reviewer #2: No

Reviewer #3: No

**********

[NOTE: If reviewer comments were submitted as an attachment file, they will be attached to this email and accessible via the submission site. Please log into your account, locate the manuscript record, and check for the action link "View Attachments". If this link does not appear, there are no attachment files.]

To ensure your figures meet our technical requirements, please review our figure guidelines: https://journals.plos.org/plosone/s/figures

You may also use PLOS’s free figure tool, NAAS, to help you prepare publication quality figures: https://journals.plos.org/plosone/s/figures#loc-tools-for-figure-preparation.

NAAS will assess whether your figures meet our technical requirements by comparing each figure against our figure specifications.

# Author comment: Author response to Decision Letter 2

**DOI:** 10.1371/journal.pone.0354338.r004

1 Jun 2026

COVER LETTER & RESPONSE TO REVIEWERS

Manuscript PONE-D-26-01733R1

"Persistence of Viable Opportunistic Pathogens in a Multi-Stage Natural Wastewater Treatment System"

Dr. Muhammad Shahid, PhD

Academic Editor, PLOS ONE

Date: 25 May 2026

Re: Manuscript PONE-D-26-01733R1 — Second Revision

Cover Letter

Dear Dr. Shahid,

We thank you and the reviewers for the constructive and thorough evaluation of our revised manuscript. We are encouraged that Reviewers #1 and #2 indicated all their previous comments have been addressed, and that Reviewer #3 considered the revised version "considerably stronger" with remaining issues mainly involving editorial cleanup, data transparency, and moderation of interpretation.

We have carefully revised the manuscript in response to all remaining comments from Reviewer #3, with particular attention to:

(i) figure numbering consistency; (ii) temporal justification of the 2015 dataset; (iii) softening of statistical claims; (iv) moderation of public-health interpretation; (v) explicit clarification of data availability; (vi) consistent terminology and hyphenation; and (vii) reduction of redundancy between Results and Discussion.

All changes are highlighted in the Revised Manuscript with Track Changes (separate file). Page and line references correspond to that document. The clean final manuscript is provided as a separate file.

We believe the manuscript is now substantially strengthened and directly addresses every point raised by Reviewer #3. We hope the revised version meets the standards for publication in PLOS ONE.

Sincerely,

Dr. Hazem Aqel (Corresponding Author)

Basic Medical Sciences Department, College of Medicine

Al-Balqa Applied University, Salt 19117, Jordan

Email: hazem.aqel@bau.edu.jo

On behalf of all co-authors

Response to Reviewers

Note: Reviewer #1 and Reviewer #2 have confirmed that all their previous comments were fully addressed. The responses below address all comments raised by Reviewer #3.

Reviewer #1 & #2

Status: All comments addressed and accepted.

Reviewer #3 — Point-by-Point Response

MAJOR ISSUES

Comment 1 — Figure numbering inconsistency

"The Methods section still refers to 'Fig 1,' although the manuscript indicates that Figure 1 was removed due to copyright concerns. Figure references should be checked carefully and updated consistently throughout the manuscript."

Response: We have removed all residual references to "Fig 1" throughout the manuscript and verified that no figure citations remain orphaned. The Methods section now describes the sampling configuration entirely in text without reference to a figure. All Table references (Tables 1–8 and Tables S1–S4) have been re-checked for consistency.

Comment 2 — Temporal limitation of the dataset (November 2015 samples)

"Adding a brief statement in the limitations section explaining the continuing value of these data would strengthen the manuscript further."

Response: We agree. We have expanded the limitations statement in both the Methods (Study Area subsection) and the Conclusions to explicitly justify the continuing value of the 2015 dataset. The added text reads:

"Although the samples analyzed in this study were collected in November 2015, the operational configuration, hydraulic residence time (4–6 d), vegetation regime, and effluent-reuse pattern of the Wadi Hanifa treatment corridor have remained substantively unchanged since that time, as confirmed through recent operational reports of the Riyadh municipal water authority. The dataset therefore retains relevance as a baseline characterization of viable opportunistic pathogen persistence in this system. Furthermore, culture-based viability data of this granularity remain scarce for arid-region nature-based treatment systems, and the dataset addresses a persistent gap in the literature regardless of collection year."

Comment 3 — Interpretation of statistical findings

"Some statements could still be softened slightly... 'declined by 50%' could be rephrased as 'declined by approximately 50%'."

Response: We have systematically softened statistical statements throughout. Key changes: Abstract: "a 50% reduction" → "an approximately 50% reduction in observed isolate richness". Results (Diversity): "Isolate richness declined by 50%..." → "Observed isolate richness declined by approximately 50%...(Cochran–Armitage exact test, p = 0.022; n = 5 stages, exploratory)". A clarifying sentence was added to the Statistical Analysis section:

"All statistical analyses presented here are exploratory and intended to identify trends consistent with biological mechanisms, rather than to provide definitive inferential conclusions."

Comment 4 — Public health interpretation (AMR reservoir/dissemination claims)

"A few statements regarding wastewater systems as reservoirs or dissemination pathways for antimicrobial resistance should still be moderated slightly."

Response: We have moderated these statements in three locations (Abstract, AMR section, Implications). The revised phrasing now reads:

"...consistent with recent reports suggesting that wastewater systems may act as environmental compartments where antimicrobial-resistant organisms can persist; however, given the limited number of isolates (n = 8) and the absence of molecular resistance gene characterization in this study, broader claims regarding dissemination cannot be made and would require dedicated genomic and longitudinal investigation."

Comment 5 — Supporting Information and raw data availability

"The authors should clarify whether the raw microbiological counts, physicochemical measurements, antimicrobial susceptibility data, and sequencing results are included within the Supporting Information files or deposited in a public repository."

Response: We have substantially revised the Data Availability Statement and added a new Supporting Information table (Table S4) containing the complete raw antimicrobial susceptibility zone-of-inhibition measurements for all eight K. pneumoniae isolates. The updated Data Availability Statement is included in full in the revised manuscript. GenBank accession numbers will be confirmed at the proof stage; a placeholder is included. We have also added explicit in-text citations to Supporting Information files at each relevant point in the Results.

MINOR ISSUES

Comment 6 — Proofreading, formatting, hyphenation

"A careful proofreading is still recommended to correct a few remaining formatting inconsistencies and typographical errors. Hyphenation and formatting should be standardized throughout the manuscript."

Response: The manuscript has been thoroughly proofread. Hyphenation is now standardized throughout: "multi-stage," "sand-filtered," "culture-based," "nature-based," and "fecal-indicator." Residual formatting inconsistencies (spacing, decimal alignment, italicization of taxonomic names) have been corrected.

Comment 7 — Terminology consistency ('viable,' 'culturable,' 'culture-based')

"Terminology should remain consistent throughout the manuscript, particularly regarding 'viable,' 'culturable,' and 'culture-based' bacteria."

Response: We have systematically standardized terminology throughout. A clarifying Terminology Note has been added to the Methods section defining each term: 'culturable' = bacteria recoverable under specific media/incubation conditions; 'viable' = synonym only insofar as culturability implies viability (with VBNC caveat); 'culture-based' = methodological framework.

Comment 8 — Statistical and decimal formatting

"Statistical reporting and decimal formatting should be checked for consistency."

Response: All p-values are now reported to three decimal places (e.g., p = 0.022, p = 0.038, p = 0.062). Correlation coefficients are reported to two decimal places. Means ± SD use consistent significant-figure conventions throughout.

Comment 9 — Repetition between Results and Discussion

"Some repetition between the Results and Discussion sections could still be reduced slightly."

Response: The Implications subsection has been condensed; it now cross-references the earlier sub-section rather than restating findings about the E. coli/K. pneumoniae contrast. Specific restatements of bacterial counts and diversity metrics have been removed from the Implications section.

Comment 10 — WHO comparison clarification

"The WHO comparison is now better qualified, but the manuscript should continue clarifying that WHO benchmarks refer to pathogen-specific reductions rather than total culturable bacterial counts."

Response: We have strengthened the clarification:

"It is important to note that WHO ≥5-log reduction targets refer to pathogen-specific reductions (e.g., for rotavirus, Cryptosporidium, and bacterial pathogens at the unit-process level), not to total culturable heterotrophic bacterial counts. The comparison here is provided solely for contextual scale; total CFU reduction is not a direct equivalent of pathogen-specific log-removal credits and should not be interpreted as such."

Comment 11 — Additional references in Results and Discussion

"The Results and Discussion sections would benefit from additional references to strengthen comparisons with previous studies."

Response: We have added six relevant references (#35–40) to strengthen comparisons on pathogen persistence, AMR in wastewater, and limitations of fecal indicators. Full citations are listed in the revised reference list.

Comment 12 — Clustering table relevance in main text

"The clustering table is informative, but a brief explanation of its relevance to pathogen persistence within the main text would improve clarity."

Response: We added the following sentence to the Results section (Shifts in viable bacterial composition subsection):

"The hierarchical clustering of biochemical profiles (Supporting Table S2) showed that all eight K. pneumoniae isolates—recovered across the full treatment train—grouped within a tight metabolic cluster distinct from E. coli, Enterobacter, and Proteus isolates, indicating phenotypic coherence of the persistent K. pneumoniae population and consistency of identification across stages."

Comment 13 — Reference list final check

"The duplicate reference issue appears to have been addressed, although the final reference list should still be checked carefully before submission."

Response: The reference list has been re-verified in full. No duplicates remain. All 40 references follow PLOS ONE Vancouver style consistently. The six new references (#35–40) have been integrated and numbered sequentially.

Summary of All Revisions

# Reviewer Concern Action Taken

1 Figure 1 references All removed; manuscript is fully figure-free; all table references re-checked

2 2015 dataset relevance New justification added to Methods (Study Area) and Conclusions

3 Statistical phrasing too strong All claims softened; exploratory framing added throughout

4 AMR reservoir/dissemination overreach Moderated in Abstract, AMR section, and Implications (3 locations)

5 Raw data availability unclear New Supporting Table S4; expanded Data Availability Statement; GenBank deposition noted

6 Proofreading / hyphenation Standardized throughout: multi-stage, sand-filtered, culture-based, nature-based

7 Terminology consistency New 'Terminology Note' added in Methods section

8 Decimal/statistical formatting Uniform 3 d.p. p-values, 2 d.p. τ values, consistent SD conventions

9 Redundancy Results/Discussion Implications section condensed; cross-references used instead of repetition

10 WHO benchmark clarification Explicit clarifying statement added

11 Additional references 6 new references (#35–40) integrated at appropriate locations

12 Clustering table relevance Explanatory sentence added in Results section

13 Reference list duplicates Re-verified — no duplicates remain

Attachment Submitted filename: Response_to_Reviewers.docx

# Peer review: Decision Letter 2

**DOI:** 10.1371/journal.pone.0354338.r005

**Authors:** Muhammad Shahid

29 Jun 2026

PONE-D-26-01733R2

Persistence of Viable Opportunistic Pathogens in a Multi-Stage Natural Wastewater Treatment System

PLOS One

Dear Dr. Aqel

Thank you for submitting your manuscript to PLOS ONE. After careful consideration, we feel that it has merit but does not fully meet PLOS ONE’s publication criteria as it currently stands. Therefore, we invite you to submit a revised version of the manuscript that addresses the points raised during the review process.

==============================

Dear Authors

The manuscript has been significantly improved; however, the reviewer suggested a couple of changes before a formal acceptance is granted.

==============================

Please submit your revised manuscript by Aug 13 2026 11:59PM. If you will need more time than this to complete your revisions, please reply to this message or contact the journal office at plosone@plos.org. When you're ready to submit your revision, log on to https://www.editorialmanager.com/pone/ and select the 'Submissions Needing Revision' folder to locate your manuscript file.

Please include the following items when submitting your revised manuscript:

- A letter that responds to each point raised by the academic editor and reviewer(s). You should upload this letter as a separate file labeled 'Response to Reviewers'.
- A marked-up copy of your manuscript that highlights changes made to the original version. You should upload this as a separate file labeled 'Revised Manuscript with Track Changes'.
- An unmarked version of your revised paper without tracked changes. You should upload this as a separate file labeled 'Manuscript'.

If you would like to make changes to your financial disclosure, please include your updated statement in your cover letter. Guidelines for resubmitting your figure files are available below the reviewer comments at the end of this letter.

If applicable, we recommend that you deposit your laboratory protocols in protocols.io to enhance the reproducibility of your results. Protocols.io assigns your protocol its own identifier (DOI) so that it can be cited independently in the future. For instructions see: https://journals.plos.org/plosone/s/submission-guidelines#loc-laboratory-protocols. Additionally, PLOS ONE offers an option for publishing peer-reviewed Lab Protocol articles, which describe protocols hosted on protocols.io. Read more information on sharing protocols at https://plos.org/protocols?utm_medium=editorial-email&utm_source=authorletters&utm_campaign=protocols.

As the corresponding author, your ORCID iD is verified in the submission system and will appear in the published article. PLOS supports the use of ORCID, and we encourage all coauthors to register for an ORCID iD and use it as well. Please encourage your coauthors to verify their ORCID iD within the submission system before final acceptance, as unverified ORCID iDs will not appear in the published article. *Only*  the individual author can complete the verification step; PLOS staff *cannot*  verify ORCID iDs on behalf of authors.

We look forward to receiving your revised manuscript.

Kind regards,

Muhammad Shahid, PhD

Academic Editor

PLOS One

Journal Requirements:

If the reviewer comments include a recommendation to cite specific previously published works, please review and evaluate these publications to determine whether they are relevant and should be cited. There is no requirement to cite these works unless the editor has indicated otherwise.

Please review your reference list to ensure that it is complete and correct. If you have cited papers that have been retracted, please include the rationale for doing so in the manuscript text, or remove these references and replace them with relevant current references. Any changes to the reference list should be mentioned in the rebuttal letter that accompanies your revised manuscript. If you need to cite a retracted article, indicate the article’s retracted status in the References list and also include a citation and full reference for the retraction notice.

Additional Editor Comments:

Dear Dr. Aqel

Thank you for making significant improvements in the manuscirpt; however, a couple of minor changes have been suggested by the reviewer before a formanl acceptance is granted.

[Note: HTML markup is below. Please do not edit.]

Reviewers' comments:

Reviewer's Responses to Questions

**Comments to the Author**

1. If the authors have adequately addressed your comments raised in a previous round of review and you feel that this manuscript is now acceptable for publication, you may indicate that here to bypass the “Comments to the Author” section, enter your conflict of interest statement in the “Confidential to Editor” section, and submit your "Accept" recommendation.

Reviewer #3: All comments have been addressed

**********

2. Is the manuscript technically sound, and do the data support the conclusions?

The manuscript must describe a technically sound piece of scientific research with data that supports the conclusions. Experiments must have been conducted rigorously, with appropriate controls, replication, and sample sizes. The conclusions must be drawn appropriately based on the data presented.

Reviewer #3: Yes

**********

3. Has the statistical analysis been performed appropriately and rigorously?

Reviewer #3: Yes

**********

4. Have the authors made all data underlying the findings in their manuscript fully available?

The PLOS Data policy requires authors to make all data underlying the findings described in their manuscript fully available without restriction, with rare exception (please refer to the Data Availability Statement in the manuscript PDF file). The data should be provided as part of the manuscript or its supporting information, or deposited to a public repository. For example, in addition to summary statistics, the data points behind means, medians and variance measures should be available. If there are restrictions on publicly sharing data—e.g. participant privacy or use of data from a third party—those must be specified.

Reviewer #3: Yes

**********

5. Is the manuscript presented in an intelligible fashion and written in standard English?

PLOS ONE does not copyedit accepted manuscripts, so the language in submitted articles must be clear, correct, and unambiguous. Any typographical or grammatical errors should be corrected at revision, so please note any specific errors here.

Reviewer #3: Yes

**********

6. Review Comments to the Author

Please use the space provided to explain your answers to the questions above. You may also include additional comments for the author, including concerns about dual publication, research ethics, or publication ethics. (Please upload your review as an attachment if it exceeds 20,000 characters)

Reviewer #3: Evaluation of the Revised Manuscript

The authors have made a substantial effort to address the comments raised during the previous round of review. Overall, the revised manuscript has improved considerably in terms of clarity, scientific interpretation, and transparency. Most of the previous concerns have been satisfactorily addressed, and the revisions have strengthened the overall quality of the manuscript. The additional explanations, revised discussion, supporting information, and updated references have improved both the readability and scientific value of the study.

Major and Minor Comments

Figure numbering – This issue has been fully addressed. Figure and table citations are now consistent throughout the manuscript.

Justification for the 2015 dataset – The authors have provided a reasonable explanation for the continued relevance of the dataset and have appropriately acknowledged its limitations.

Interpretation of the statistical analyses – The revised manuscript now presents the statistical findings more cautiously, and the exploratory nature of the analyses is clearly stated. This significantly improves the interpretation of the results.

Public health implications – The discussion has been revised appropriately, and statements regarding antimicrobial resistance dissemination are now better balanced and supported by the presented data.

Data availability and supporting information – The expanded Data Availability Statement and the inclusion of the supporting tables improve the transparency and reproducibility of the study.

Language and formatting – The manuscript has been carefully revised, and the overall language, formatting, and presentation have improved considerably.

Table presentation – Although the tables are well organized and their titles are appropriate, I recommend adding footnotes below each table to define all abbreviations used. For example, abbreviations such as n (sample size), CFU (colony-forming units), PA (Pseudomonas aeruginosa), % (percentage), and any other abbreviations should be explained beneath the corresponding table. This will make each table self-contained and easier for readers to interpret without referring back to the main text.

Terminology – I appreciate the addition of the terminology note. However, the statement indicating that "viable" is used as a synonym for "culturable" should be reconsidered. From a microbiological perspective, recovery by culture provides evidence of viability under the specific experimental conditions, but the two terms are not synonymous because viable-but-non-culturable (VBNC) bacteria remain viable despite not being recoverable by conventional culture methods. A slight revision of this wording would improve the scientific accuracy of the manuscript.

Statistical reporting – The statistical notation and formatting are now consistent and appropriate.

Results and Discussion – The reduction of repetitive information has improved the flow of the manuscript, and the Discussion is now more concise.

WHO comparison – The clarification regarding WHO log-reduction targets is appropriate and helps avoid possible misinterpretation.

Literature update – The inclusion of recent references strengthens the discussion and provides better context for the findings.

Reference formatting – The reference list has been improved substantially. However, before final acceptance, I recommend performing one final review to ensure that all references fully comply with the PLOS ONE formatting guidelines. In particular, the authors should verify the formatting of author lists, the appropriate use of et al., journal abbreviations, article numbers, DOI formatting (where applicable), and overall consistency across the reference list.

Supporting figures – If available, I encourage the authors to include representative figures supporting the molecular findings, such as agarose gel electrophoresis images of PCR products or other relevant laboratory images, either in the main manuscript or as Supporting Information. Although this is not essential for acceptance, such figures would further enhance the transparency of the study and provide additional support for the reported molecular results.

Overall Recommendation

Overall, I believe the authors have responded well to the previous review, and the manuscript has improved substantially. The remaining issues are minor and mainly concern editorial presentation and scientific precision rather than the validity of the study itself.

Before final acceptance, I recommend that the authors:

Refine the terminology distinguishing viable and culturable bacteria to better reflect accepted microbiological concepts.

Add explanatory footnotes defining all abbreviations used in each table (e.g., n = sample size, CFU = colony-forming units, PA = Pseudomonas aeruginosa, % = percentage, and other abbreviations where applicable).

Perform a final check of all references to ensure complete compliance with the PLOS ONE reference formatting guidelines.

If available, include representative supporting figures (e.g., agarose gel electrophoresis images of PCR products or other relevant laboratory images) to further improve the transparency and documentation of the experimental findings.

These minor revisions will further improve the clarity, readability, and presentation of the manuscript without affecting scientific findings or conclusions.

Recommendation: Accept after Minor Revision.

**********

7. PLOS authors have the option to publish the peer review history of their article (what does this mean?). If published, this will include your full peer review and any attached files.

If you choose “no”, your identity will remain anonymous but your review may still be made public.

**Do you want your identity to be public for this peer review?**  For information about this choice, including consent withdrawal, please see our Privacy Policy.

Reviewer #3: No

**********

[NOTE: If reviewer comments were submitted as an attachment file, they will be attached to this email and accessible via the submission site. Please log into your account, locate the manuscript record, and check for the action link "View Attachments". If this link does not appear, there are no attachment files.]

To ensure your figures meet our technical requirements, please review our figure guidelines: https://journals.plos.org/plosone/s/figures

You may also use PLOS’s free figure tool, NAAS, to help you prepare publication quality figures: https://journals.plos.org/plosone/s/figures#loc-tools-for-figure-preparation.

NAAS will assess whether your figures meet our technical requirements by comparing each figure against our figure specifications.

# Author comment: Author response to Decision Letter 3

**DOI:** 10.1371/journal.pone.0354338.r006

4 Jul 2026

Manuscript: PONE-D-26-01733R2

Title: "Persistence of Viable Opportunistic Pathogens in a Multi-Stage Natural Wastewater Treatment System"

Response to Reviewer #3:

We thank Reviewer #3 for the careful and constructive evaluation of our revised manuscript. We are encouraged that the reviewer found the manuscript "improved considerably" and recommended acceptance after minor revision. We have addressed each of the remaining comments in detail below.

Review Summary

We appreciate the reviewer's overall positive assessment that "the authors have made a substantial effort to address the comments raised during the previous round of review" and that "the revised manuscript has improved considerably in terms of clarity, scientific interpretation, and transparency."

We are grateful for the recognition that our revisions have "strengthened the overall quality of the manuscript" and that "the additional explanations, revised discussion, supporting information, and updated references have improved both the readability and scientific value of the study."

Responses to Specific Comments

Terminology (Comment 1)

Reviewer Comment: "I appreciate the addition of the terminology note. However, the statement indicating that 'viable' is used as a synonym for 'culturable' should be reconsidered. From a microbiological perspective, recovery by culture provides evidence of viability under the specific experimental conditions, but the two terms are not synonymous because viable-but-non-culturable (VBNC) bacteria remain viable despite not being recoverable by conventional culture methods. A slight revision of this wording would improve the scientific accuracy of the manuscript."

Response: We agree with the reviewer's important distinction and have revised the Terminology Note to more accurately reflect the relationship between viability and culturability. The revised note now reads:

Terminology Note. Throughout this manuscript, "culturable" refers to bacteria recoverable under the specific media and incubation conditions used in this study. "Viable" is used in the conventional sense of having metabolic activity and the potential for growth; however, culturability is considered a proxy for viability rather than a perfect synonym, as the viable-but-non-culturable (VBNC) state is well documented for many bacterial taxa, including Klebsiella pneumoniae, and is not captured by the culture-based approach used here. "Culture-based" describes the overall methodological framework, and findings should be interpreted with the understanding that VBNC cells are not enumerated.

[Location: Materials and Methods, Study Area and Sampling Design subsection]

Table Footnotes (Comment 2)

Reviewer Comment: "Although the tables are well organized and their titles are appropriate, I recommend adding footnotes below each table to define all abbreviations used. For example, abbreviations such as n (sample size), CFU (colony-forming units), PA (Pseudomonas aeruginosa), % (percentage), and any other abbreviations should be explained beneath the corresponding table. This will make each table self-contained and easier for readers to interpret without referring back to the main text."

Response: We have added comprehensive footnotes to all tables in the manuscript and Supporting Information to define all abbreviations used. The footnotes have been added as follows:

Table 1 (Physicochemical parameters):

Footnote: Values are mean ± standard deviation from triplicate replicate measurements at each sampling location (n = 3). DO = dissolved oxygen; EC = electrical conductivity; NTU = nephelometric turbidity units; Temp = temperature; °C = degrees Celsius; mg/L = milligrams per liter; μS/cm = microsiemens per centimeter.

Table 2 (Culturable bacterial counts):

Footnote: Values are mean ± standard deviation from triplicate plate counts (n = 3 plates per dilution). BA = blood agar; MAC = MacConkey agar; CFU = colony-forming units; mL = milliliter. The highest dilution yielding countable colonies was used for enumeration in each case.

Table 3 (Bacterial isolates identification):

Footnote: Presumptive identification based on API 20E biochemical profiles (bioMérieux, Marcy-l'Étoile, France). API codes are 7-digit numerical profiles; identification confidence based on manufacturer's database (≥85% considered acceptable). n = number of isolates; % = percentage of total isolates characterized; L3.1-L3.5 = sampling locations along treatment train (influent to final effluent). Isolate IDs correspond to morphologically distinct colonies selected from blood agar and MacConkey agar plates.

Table 4 (Antimicrobial susceptibility patterns):

Footnote: Antimicrobial susceptibility testing performed by Kirby-Bauer disk diffusion on Mueller-Hinton agar following CLSI M100 (33rd edition, 2023) guidelines. n = 8 K. pneumoniae isolates recovered across all treatment stages. MDR = multidrug resistance, defined as resistance to ≥3 antimicrobial classes. Percentages are based on n = 8 isolates. CLSI interpretive criteria: S = susceptible, I = intermediate, R = resistant. All isolates were tested in triplicate (raw zone-of-inhibition measurements provided in Supporting Table S4).

Table 5 (Bacterial diversity metrics):

Footnote: S = observed isolate richness (number of distinct morphotypes isolated per 500 mL sample). H' = Shannon-Wiener diversity index. 1-D = Simpson's diversity index. J' = Pielou's evenness (H'/ln S). Values are based on 18 morphologically distinct isolates characterized across five treatment stages. L3.1-L3.5 = sequential treatment stages from influent to final effluent.

Table 6 (Kendall's τ correlations):

Footnote: τ = Kendall's rank correlation coefficient; p = p-value (two-tailed). n = 5 treatment stages. *Significant at α = 0.05. DO = dissolved oxygen; EC = electrical conductivity. All p-values are reported as exploratory given the small sample size. Interpretations are qualitative and intended to identify trends consistent with biological mechanisms.

Table 7 (Molecular characterization of representative K. pneumoniae isolates):

Footnote: %G+C = guanine-plus-cytosine content determined by thermal denaturation method (Marmur and Doty, 1962). Values are mean ± standard deviation from triplicate thermal denaturation measurements. bp = base pairs. PCR = polymerase chain reaction. All five isolates confirmed as consistent with Klebsiella pneumoniae based on %G+C content (expected range: 57-58%) and 16S rRNA PCR amplicon size (expected size: ~657 bp). See Supporting Table S4 for raw agarose gel images.

[Location: Beneath each table in the main manuscript]

Reference Formatting (Comment 3)

Reviewer Comment: "The reference list has been improved substantially. However, before final acceptance, I recommend performing one final review to ensure that all references fully comply with the PLOS ONE formatting guidelines. In particular, the authors should verify the formatting of author lists, the appropriate use of et al., journal abbreviations, article numbers, DOI formatting (where applicable), and overall consistency across the reference list."

Response: We have performed a final comprehensive review of the reference list to ensure full compliance with PLOS ONE formatting guidelines. Specific checks conducted:

1. Author lists: Verified that for references with ≤6 authors, all authors are listed; for references with >6 authors, the first 6 are listed followed by "et al." This has been applied consistently.

2. Journal abbreviations: Standard ISO abbreviations have been used for all journals (e.g., Water Res, Sci Total Environ, PLoS One).

3. Article numbers: Where provided, article numbers (e.g., e0309409) are included and formatted correctly.

4. DOI formatting: DOIs have been added where available and formatted as hyperlinks.

5. Reference order: All references are numbered sequentially in the order of first citation in the text and are listed in the same order in the reference list.

6. Consistency: All references now follow a uniform format with respect to author names, article titles, journal names, volume, issue, pages, and year.

The reference list has been re-verified, and no duplicates remain. All 40 references are now fully compliant with PLOS ONE Vancouver style.

[Location: Reference list, pages 26-28]

Supporting Figures (Comment 4)

Reviewer Comment: "If available, I encourage the authors to include representative figures supporting the molecular findings, such as agarose gel electrophoresis images of PCR products or other relevant laboratory images, either in the main manuscript or as Supporting Information. Although this is not essential for acceptance, such figures would further enhance the transparency of the study and provide additional support for the reported molecular results."

Response: We agree with the reviewer that including representative molecular figures enhances transparency. We have prepared Supporting Figure S1 containing:

1. Representative agarose gel electrophoresis image showing 16S rRNA PCR products (~657 bp) for five K. pneumoniae isolates (one from each treatment stage), together with positive (K. pneumoniae ATCC 13883) and negative (sterile water) controls.

2. A separate gel image showing a subset of PCR products with a 100 bp DNA ladder for size verification.

These figures have been added as Supporting Figure S1, with an accompanying figure legend describing the experimental conditions and interpretation. The figure is cited in the manuscript text at the appropriate location (Molecular Characterization subsection) and is included in the Supporting Information file.

[Location: Supporting Information; cited in Results and Discussion, Molecular Characterization subsection]

Summary of Revisions

# Reviewer Concern Action Taken

1 Terminology: viable vs. culturable distinction Revised Terminology Note to explicitly address VBNC caveat; clarified that culturability is a proxy rather than synonym for viability

2 Table abbreviations undefined Added comprehensive footnotes to all tables (Tables 1-7) defining abbreviations (n, CFU, PA, %, etc.)

3 Reference formatting verification Performed final check of all 40 references for compliance with PLOS ONE Vancouver style; verified author lists, journal abbreviations, DOIs, consistency

4 Supporting molecular figures Added Supporting Figure S1 with agarose gel images of 16S rRNA PCR products; included figure legend

Additional Editorial Revisions

In addition to the specific reviewer-requested changes, we have made the following minor corrections:

1. Scientific precision: Throughout the manuscript, we have refined language to ensure that claims are appropriately qualified and that the exploratory nature of the study is consistently communicated.

2. Formatting consistency: We have verified that all scientific nomenclature is correctly italicized (e.g., Escherichia coli, Klebsiella pneumoniae), that unit abbreviations are standardized (e.g., mL, CFU, mg/L), and that decimal alignments are consistent.

3. Figure citation: Supporting Figure S1 has been cited appropriately in the text where molecular results are discussed.

4. Supporting Information: The Supporting Information file has been updated to include the new Supporting Figure S1 and to verify that all supporting tables are correctly referenced in the main text.

Conclusion

We are grateful for the thorough and constructive review provided by Reviewer #3. All remaining comments have been addressed in this revision, and we believe the manuscript is now ready for final acceptance in PLOS ONE. We hope that the reviewers and editors find our responses satisfactory and that the manuscript meets the journal's high standards for publication.

Sincerely,

Dr. Hazem Aqel (Corresponding Author)

Basic Medical Sciences Department

College of Medicine, Al-Balqa Applied University

Salt 19117, Jordan

Email: hazem.aqel@bau.edu.jo

On behalf of all co-authors

Attachment Submitted filename: Response_to_Reviewers_auresp_3.docx

# Editor report: Decision Letter 3

**DOI:** 10.1371/journal.pone.0354338.r007

**Authors:** Muhammad Shahid

8 Jul 2026

Persistence of Viable Opportunistic Pathogens in a Multi-Stage Natural Wastewater Treatment System

PONE-D-26-01733R3

Dear Dr. Aqel

We’re pleased to inform you that your manuscript has been judged scientifically suitable for publication and will be formally accepted for publication once it meets all outstanding technical requirements.

Within one week, you’ll receive an e-mail detailing the required amendments. When these have been addressed, you’ll receive a formal acceptance letter and your manuscript will be scheduled for publication.

An invoice will be generated when your article is formally accepted. Please note, if your institution has a publishing partnership with PLOS and your article meets the relevant criteria, all or part of your publication costs will be covered. Please make sure your user information is up-to-date by logging into Editorial Manager at Editorial Manager® and clicking the ‘Update My Information' link at the top of the page. For questions related to billing, please contact billing support.

If your institution or institutions have a press office, please notify them about your upcoming paper to help maximize its impact. If they’ll be preparing press materials, please inform our press team as soon as possible -- no later than 48 hours after receiving the formal acceptance. Your manuscript will remain under strict press embargo until 2 pm Eastern Time on the date of publication. For more information, please contact onepress@plos.org.

Kind regards,

Muhammad Shahid, PhD

Academic Editor

PLOS One

Additional Editor Comments (optional):

Reviewers' comments:

# Editor report: Acceptance letter

**DOI:** 10.1371/journal.pone.0354338.r008

**Authors:** Muhammad Shahid

PONE-D-26-01733R3

PLOS One

Dear Dr. Aqel,

I'm pleased to inform you that your manuscript has been deemed suitable for publication in PLOS One. Congratulations! Your manuscript is now being handed over to our production team.

At this stage, our production department will prepare your paper for publication. This includes ensuring the following:

* All references, tables, and figures are properly cited

* All relevant supporting information is included in the manuscript submission,

* There are no issues that prevent the paper from being properly typeset

You will receive further instructions from the production team, including instructions on how to review your proof when it is ready. Please keep in mind that we are working through a large volume of accepted articles, so please give us a few days to review your paper and let you know the next and final steps.

Lastly, if your institution or institutions have a press office, please let them know about your upcoming paper now to help maximize its impact. If they'll be preparing press materials, please inform our press team within the next 48 hours. Your manuscript will remain under strict press embargo until 2 pm Eastern Time on the date of publication. For more information, please contact onepress@plos.org.

You will receive an invoice from PLOS for your publication fee after your manuscript has reached the completed accept phase. If you receive an email requesting payment before acceptance or for any other service, this may be a phishing scheme. Learn how to identify phishing emails and protect your accounts at https://explore.plos.org/phishing.

If we can help with anything else, please email us at customercare@plos.org.

Thank you for submitting your work to PLOS ONE and supporting open access.

Kind regards,

PLOS ONE Editorial Office Staff

on behalf of

Dr. Muhammad Shahid

Academic Editor

PLOS One
